# FBXL2 counteracts Grp94 to destabilize EGFR and inhibit EGFR-driven NSCLC growth

**DOI:** 10.1038/s41467-021-26222-x

**Published:** 2021-10-11

**Authors:** Mengmeng Niu, Jing Xu, Yang Liu, Yuhuang Li, Tao He, Liangping Ding, Yajun He, Yong Yi, Fengtian Li, Rongtian Guo, Ya Gao, Rui Li, Luping Li, Mengyuan Fu, Qingyong Hu, Yangkun Luo, Chunyan Zhang, Kewei Qin, Jianqiao Yi, Shuhan Yu, Jian Yang, Hu Chen, Liang Wang, Zhonghan Li, Biao Dong, Shiqian Qi, Liang Ouyang, Yujun Zhang, Yang Cao, Zhi-Xiong Jim Xiao

**Affiliations:** 1 Center of Growth, Metabolism, and Aging, Key Laboratory of Bio-Resource and Eco-Environment, Ministry of Education, College of Life Sciences, Sichuan University, Chengdu, China; 2 State Key Laboratory of Biotherapy, West China Hospital, Sichuan University, Chengdu, China

**Keywords:** Non-small-cell lung cancer, Growth factor signalling, Ubiquitylation

## Abstract

Abnormal activation of epidermal growth factor receptor (EGFR) drives non-small cell lung cancer (NSCLC) development. EGFR mutations-mediated resistance to tyrosine-kinase inhibitors (TKIs) is a major hurdle for NSCLC treatment. Here, we show that F-box protein FBXL2 targets EGFR and EGFR TKI-resistant mutants for proteasome-mediated degradation, resulting in suppression of EGFR-driven NSCLC growth. Reduced FBXL2 expression is associated with poor clinical outcomes of NSCLC patients. Furthermore, we show that glucose-regulated protein 94 (Grp94) protects EGFR from degradation via blockage of FBXL2 binding to EGFR. Moreover, we have identified nebivolol, a clinically used small molecule inhibitor, that can upregulate FBXL2 expression to inhibit EGFR-driven NSCLC growth. Nebivolol in combination with osimertinib or Grp94-inhibitor-1 exhibits strong inhibitory effects on osimertinib-resistant NSCLC. Together, this study demonstrates that the FBXL2-Grp94-EGFR axis plays a critical role in NSCLC development and suggests that targeting FBXL2-Grp94 to destabilize EGFR may represent a putative therapeutic strategy for TKI-resistant NSCLC.

## Abstract (web summary)

Aberrant EGFR activation is commonly found in non-small cell lung cancer (NSCLC). Here the authors show that E3 ubiquitin ligase FBXL2 targets EGFR and EGFR tyrosine kinase inhibitor (TKI)-resistant mutants for proteasome-mediated degradation to inhibit EGFR-driven NSCLC growth and TKI resistance.

## Introduction

Epidermal growth factor receptor (EGFR) is overexpressed or aberrantly activated in various human cancers, including cancers in lung, head and neck, colon, and brain^1–3^. Dysregulation of EGFR protein stability is shown to critically contribute to abnormal EGFR signaling and cancer development^4–6^. It is therefore fundamentally important in elucidating the molecular mechanisms by which EGFR protein stability is controlled. It has been shown that EGFR stability is tightly regulated upon EGF stimulation. E3 ubiquitin ligase c-Cbl can induce EGFR mono-ubiquitination, endocytosis, and lysosome-mediated degradation, resulting in attenuation of EGFR signaling upon EGF stimulation^7,8^. Although proteasome pathway is also involved in EGFR degradation^9^, the exact mechanisms underlying proteasome-dependent degradation of EGFR are largely unclear.

Activation of EGFR signaling, including *EGFR* gene amplification and occurrence of activation mutations, such as L858R and exon 19 deletions, is a major driving force for non-small-cell lung cancer (NSCLC), which accounts for approximately 85% of all lung cancer cases^10,11^. Targeted therapy via EGFR tyrosine kinase inhibitors (TKIs), such as erlotinib or gefitinib, has been widely used for the treatment of patients with EGFR-activating mutations with greatly improved patient outcomes^12^. However, the vast majority of NSCLC patients have acquired resistance to EGFR-TKIs, in which the EGFR^T790M^ mutation accounts for approximately 50% of all 1st-generation TKIs resistant cases^13^. Osimertinib (AZD9291) has been developed to overcome EGFR^T790M^-mediated TKI resistance. However, new acquired *EGFR* resistant mutations to osimertinib have been also emerged relentlessly, including EGFR^C797S^ and EGFR^L718Q^ ^14,15^. Therefore, new strategies to combat TKI-resistant NSCLC are urgently needed.

Glucose-regulated protein 94 (Grp94) is an endoplasmic reticulum (ER) resident member of the heat shock protein 90 (Hsp90) family involved in protein homeostasis^16^. Grp94 functions as a chaperone to direct folding and/or assembly of several secreted and membrane proteins, including immunoglobulin (Ig) family, bile salt-dependent lipase (BSDL), Toll-like receptor, and integrins^17^. Grp94 has been shown to play an important role in tumor growth and metastasis in a variety of cancers, including lung cancer, melanoma, ovarian cancer, and multiple myeloma^18,19^. Clinically, Grp94 is frequently overexpressed in advanced cancers with poor survival^20,21^. However, the role of Grp94 on EGFR protein homeostasis is currently unknown.

The E3 ubiquitin ligase FBXL2 is a member of the conserved F-box family proteins that determine the specificity of SCF ubiquitin ligase complex^22^. FBXL2 possesses a unique CaaX motif at the C-terminus essential for geranylgeranylation and membrane targeting^23^. FBXL2 can promote proteasomal degradation of cyclin D2, cyclin D3, Aurora B, IP3R3, or p85β resulting in inhibition of cell proliferation or suppression of apoptosis/autophagy^24–28^. FBXL2 can also target TRAF proteins for degradation to regulate inflammation^26^. In this study, we demonstrate that FBXL2 is an E3 ubiquitin ligase targeting EGFR and EGFR TKI-resistant mutants for proteasome-mediated degradation to inhibit EGFR-driven NSCLC growth. Notably, FBXL2 can compete with Grp94 for EGFR binding. Activation of FBXL2, as exemplified by nebivolol identified in this study, in combination with Grp94-inhibitor exhibits strong inhibitory effects on osimertinib-resistant NSCLC.

## Results

### FBXL2 binds to and promotes EGFR for polyubiquitin- and proteasome-mediated degradation in an EGF-independent manner

EGFR protein can be rapidly destabilized through c-Cbl-mediated lysosome pathway upon EGF stimulation^7,8^. Since EGFR protein levels are critically important in tumorigenesis, we reasoned that EGFR protein stability ought to be tightly controlled at multiple levels. Thus, we examined the impact of proteasome-mediated EGFR protein degradation with or without EGF induction. As shown in Supplementary Fig. 1a, EGF-induced EGFR degradation was blocked by either a proteasome inhibitor (MG132) or a lysosome inhibitor (chloroquine, CLQ) in NSCLC H1299 (wild-type EGFR) or H1975 (EGFR^L858R/T790M^) cells. By contrast, in the absence of EGF induction, EGFR protein degradation was primarily via proteasome pathway, suggesting that proteasome-mediated degradation is critically important in the regulation of EGFR protein stability. Thus, we aimed to screen for E3 ubiquitin ligase(s) targeting EGFR for proteasomal degradation using a lentiviral-based shRNA library specific for E3 ubiquitin ligases.

**Fig. 1:**
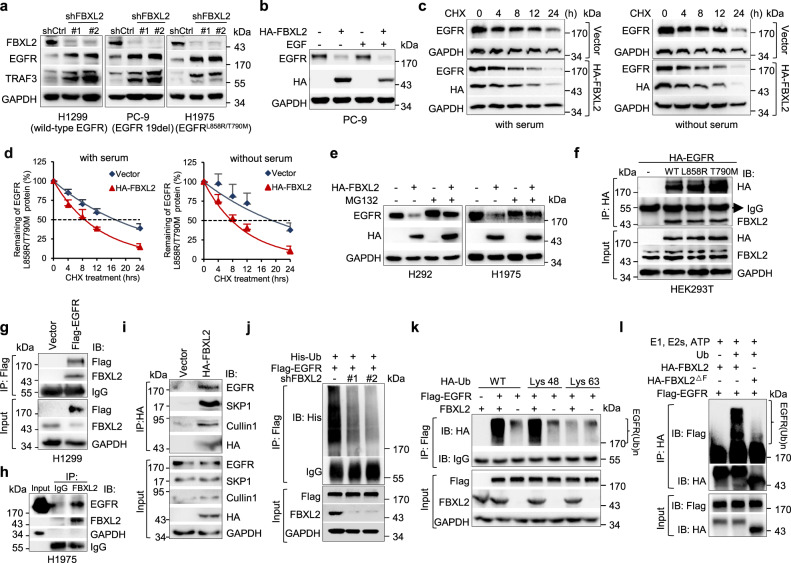
FBXL2 targets EGFR for polyubiquitin- and proteasome-mediated degradation in an EGF-independent manner. **a** H1299, PC-9, or H1975 cells stably expressing different shRNAs against FBXL2 (shFBXL2-#1 or shFBXL2-#2) or GFP (shCtrl) were subjected to Western blot analyses. **b** PC-9 cells stably expressing HA-FBXL2 were grown in serum-free medium for 12 h, prior to treatment with or without 100 ng/mL EGF for 10 min. Cell lysates were subjected to Western blot analyses. **c**, **d** H1975 cells stably expressing HA-FBXL2 were grown in the absence or presence of serum (10%) for 12 h, prior to the treatment with 50 µg/mL cycloheximide (CHX) for an indicated time interval. Cell lysates were subjected to Western blot analyses. Three independent experiments were performed. The EGFR protein levels were quantified and a plot representing protein half-life was presented as means ± SD. **e** H292 or H1975 cells stably expressing HA-FBXL2 were treated with MG132 for 6 h prior to Western blot analyses. **f** HEK293T cells were co-transfected with FBXL2 and an indicated EGFR expressing plasmids. Cells were grown overnight and treated with 20 µM MG132 for 4 h, followed by IP (immunoprecipitation)-Western analyses. **g** H1299 cells stably expressing Flag-EGFR were treated with MG132 for 4 h before harvesting for IP-Western analyses. **h** H1975 cells were treated with MG132 for 4 h, and endogenous FBXL2 protein was immunoprecipitated with a specific antibody for FBXL2 or normal rabbit IgG, followed by immunoblotting. **i** H1299 cells stably expressing HA-FBXL2 were treated with MG132 for 4 h before harvesting for IP-Western analyses. **j** HEK293T cells were co-transfected with indicated expressing plasmids. Cells were grown overnight and treated with MG132 for 4 h, followed by IP-Western analyses. **k** HEK293T cells were co-transfected with Flag-EGFR, FBXL2, and either HA-ubiquitin wild-type, Lys 48-only, or Lys 63-only expressing plasmids. Cells were grown overnight and treated with MG132 for 4 h, followed by IP-Western analyses. **l** In vitro ubiquitination reaction mixtures were subjected to immunoblotting with either antibody specific for Flag or HA. The experiment was repeated three times independently with similar results (**a**, **b**, **e**–**l**). Source data are provided as a Source data file.

Among 22 candidate genes of the F-box protein family screened, silencing of FBXL2 markedly and reproducibly upregulated protein expression of wild-type EGFR, EGFR 19del, or EGFR^L858R/T790M^ in H1299, PC-9 or H1975 cells, respectively (Supplementary Fig. 1b, c and Fig. 1a). TRAF3, a known substrate of FBXL2^26^, was analyzed in parallel. Conversely, ectopic expression of FBXL2 led to downregulation of EGFR protein expression (Supplementary Fig. 1d). By contrast, expression of FBXL2 mutant defective in E3 ubiquitin ligase activity, including FBXL2^ΔF^ (deletion of F-box domain) or FBXL2^4A^ (four-point mutations in the F-box domain)^27^, failed to do so (Supplementary Fig. 3e and Supplementary Fig. 4b). These results indicate that FBXL2 can inhibit EGFR expression, which is dependent on its E3 ubiquitin ligase activity.

We then investigated whether EGF stimulation is required for FBXL2-mediated EGFR degradation. As shown in Fig. 1b, FBXL2 effectively degraded EGFR either in the presence or absence of EGF in PC-9 cells. Similar effects were observed in H1299 and H1975 cells (Supplementary Fig. 1e). In addition, FBXL2 significantly shortened the half-life of EGFR^L858R/T790M^ protein, regardless of the presence or absence of serum (Fig. 1c, d). Therefore, FBXL2-mediated EGFR protein degradation is independent of EGF stimulation.

We next investigated whether FBXL2 is a bona fide E3 ubiquitin ligase for EGFR. As shown in Fig. 1e and Supplementary Fig. 1f, g, FBXL2-mediated reduction of EGFR protein levels was effectively reversed by proteasome inhibitor MG132, but not by lysosome inhibitor chloroquine. Furthermore, FBXL2 interacted with wild-type EGFR, EGFR^L858R^, or EGFR^T790M^ protein and FBXL2 formed stable complexes with EGFR, SKP1, and Cullin1 (Fig. 1f−i), suggesting that the SCF^FBXL2^ complex is responsible for EGFR protein degradation. Moreover, silencing of FBXL2 led to reduced polyubiquitin chains of EGFR protein, whereas ectopic expression of FBXL2 facilitated Lys 48-linked, but not Lys 63-linked, polyubiquitin chains of EGFR protein (Fig. 1j, k). Immunopurified FBXL2, but not FBXL2^ΔF^, promoted in vitro ubiquitination of EGFR (Fig. 1l). Thus, FBXL2 binds to and promotes polyubiquitin of EGFR, resulting in proteasome-mediated degradation of EGFR.

### FBXL2 targets on the membrane and binds to the kinase domain of EGFR to promote EGFR protein degradation

**Fig. 2:**
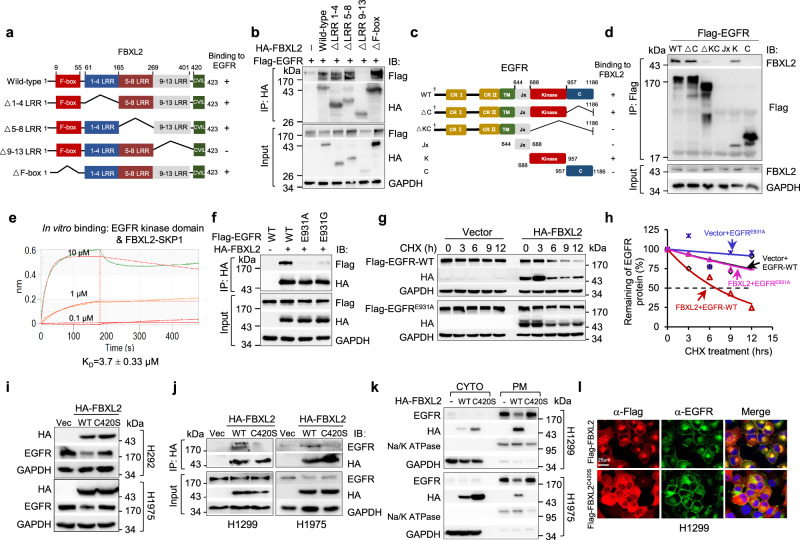
FBXL2 interacts with the kinase domain of EGFR and its membrane-targeting function is essential for the destabilization of EGFR protein. **a** A schematic representation of FBXL2 deletion mutants used in this study. **b** HEK293T cells were co-transfected with Flag-EGFR and an indicated HA-FBXL2 expressing plasmids. After grown overnight, cells were treated with MG132 for 4 h before harvesting for IP-Western analyses. **c** A schematic representation of EGFR deletion mutants constructed in this study. **d** HEK293T cells were co-transfected with FBXL2 and an indicated Flag-EGFR expressing plasmids. After grown overnight, cells were treated with MG132 for 4 h before harvesting for IP-Western analyses. **e** The binding affinity of the recombinant FBXL2/SKP1 protein and EGFR kinase portion was measured by Biolayer interferometry (BLI) assay. The *K*_D_ values were shown. **f** HEK293T cells were co-transfected with HA-FBXL2 and indicated Flag-EGFR expressing plasmids. Cells were grown overnight and treated with MG132 for 4 h, followed by IP-Western analyses. **g**, **h** HEK293T cells were co-transfected with wild-type Flag-EGFR or Flag-EGFR^E931A^ in the presence of HA-FBXL2 or a vector control for 36 h. Cells were then treated with 50 µg/mL cycloheximide (CHX) for an indicated time interval. Cell lysates were subjected to Western blot analyses. The EGFR protein levels were quantified and a plot representing protein half-life was presented. **i** H292 or H1975 cells stably expressing HA-FBXL2 or HA-FBXL2^C420S^ were subjected to Western blot analyses. **j** H1299 or H1975 cells stably expressing HA-FBXL2, HA-FBXL2^C420S^, or a vector control were treated with MG132 for 4 h before harvesting for IP-Western analyses. **k** H1299 or H1975 cells stably expressing HA-FBXL2 or HA-FBXL2^C420S^ were subjected to fractionation of cytoplasmic (CYTO) and plasma membrane (PM), followed by Western blot analyses. **l** H1299 cells stably expressing Flag-FBXL2 or Flag-FBXL2^C420S^ were treated with MG132 for 6 h prior to immunostaining for Flag (Red) and endogenous EGFR (Green) and counterstained with DAPI. Scale bar = 25 µm. The experiment was repeated three times independently with similar results (**b**, **d**, **f**, **g**, and **i**−**l**). Source data are provided as a Source data file.

We next mapped the FBXL2-binding domain on EGFR. As shown in Fig. 2a, b, the section of 9−13 leucine-rich repeat (LRR) of FBXL2 (aa 269-401) bound to EGFR. Furthermore, the kinase domain of EGFR (aa 688-957) was necessary and sufficient to interact with FBXL2 (Fig. 2c, d). Importantly, the purified recombinant FBXL2-SKP1 protein complex was able to directly bind to the kinase domain of EGFR in vitro, as evidenced by Biolayer Interferometry assay (Fig. 2e and Supplementary Fig. 2a).

We then dissected the potential FBXL2-binding site(s) in the EGFR kinase domain. We employed ZDOCK 3.0.2 (https://zdock.umassmed.edu/), a frequently used prediction tool for protein−protein interaction. As shown in Supplementary Fig. 2b, there are seven potential amino acid residues (Ser720, Lys806, Asp807, Lys875, Ser921, Glu922, and Glu931) on the interface of the EGFR kinase domain, which may be involved in interaction with FBXL2 through hydrogen bonds. We then examined the FBXL2-binding ability of EGFR mutant protein on these seven residues. Interestingly, except for EGFR^E931A^ that was unable to bind FBXL2, other mutant proteins bound to FBXL2 similar to that of wild-type EGFR (Supplementary Fig. 2c). Notably, it has been reported that EGFR^E931G^ is resistant to EGFR-TKIs including erlotinib in vitro^29^. When tested, EGFR^E931G^ was also unable to bind FBXL2 (Fig. 2f). Indeed, in sharp contrast to wild-type EGFR, FBXL2 had little effects on either expression or stability of EGFR^E931A^ or EGFR^E931G^ protein (Supplementary Fig. 2d and Fig. 2g, h). These results indicate that Glu931 in the EGFR kinase domain is indispensable for its interaction with FBXL2.

It has been documented that FBXL2 can be targeted to membranes through geranyl-geranylated Cys 420 in the CaaX motif at the C-termini^27,28^. We then asked whether the membrane targeting of FBXL2 is prerequisite for its effects on EGFR expression. As shown in Fig. 2i, j, unlike wild-type FBXL2, FBXL2^C420S^, a mutant protein defective in targeting to membrane^27^, was unable to bind to and inhibit expression of EGFR protein in various NSCLC cells. In addition, cell fractionation experiments showed that plasma membrane-associated FBXL2, but not cytosolic FBXL2^C420S^, induced degradation of plasma membrane-associated EGFR (Fig. 2k). Furthermore, ectopically expressed FBXL2 was co-localized with EGFR and reduced expression of EGFR on the plasma membrane, as evidenced by immunofluorescence and flow cytometry analyses (Fig. 2l and Supplementary Fig. 2e). Interestingly, EGFR and FBXL2 were also co-localized on the endoplasmic reticulum (ER), which was further confirmed by staining of either an ER tracker or glucose-regulated protein 78 (Grp78), a frequently used ER marker^30^ (Fig. 2l and Supplementary Fig. 2f). In keeping with this observation, FBXL2, but not FBXL2^C420S^, significantly decreased both plasma membrane-associated EGFR and ER-associated EGFR (Supplementary Fig. 2g, h). Thus, the membrane-targeting function of FBXL2 is essential for EGFR protein instability on both plasma membrane and ER.

### FBXL2 inhibits EGFR-overexpressed or EGFR^L858R/T790M^-driven NSCLC growth and is reversely correlated with EGFR expression in NSCLC

**Fig. 3:**
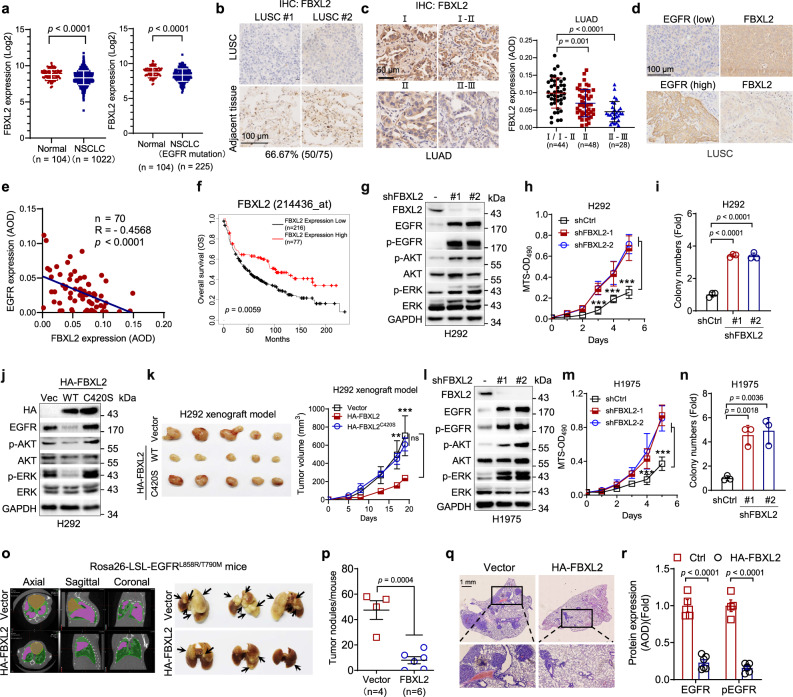
FBXL2 inhibits EGFR-overexpressed or EGFR^L858R/T790M^-driven NSCLC growth and the expression of FBXL2 and EGFR is reversely correlated. **a** The mRNA levels of FBXL2 in NSCLC (*n* = 1022) or NSCLC with EGFR mutations (*n* = 225) and normal lung tissues (*n* = 104) were analyzed using the TCGA database (LUAD and LUSC). **b** Tissue microarray slides derived from LUSC (*n* = 75 pairs of tumors and adjacent samples) were subjected to IHC analysis. Representative images of IHC staining were shown. **c** Tissue microarray slides derived from LUAD (*n* = 120) were subjected to IHC analysis. Representative images of IHC staining were shown. Staining was quantified by AOD. **d**, **e** Consecutive tissue microarray slides derived from LUSC were subjected to IHC assay for EGFR and FBXL2 expression and analyzed for Pearson correlation. Staining was quantified by AOD. **f** Kaplan−Meier plots of overall survival (OS) of human lung cancer patients were stratified by the FBXL2 mRNA levels in the patient tumor samples. H292 cells stably expressing shRNAs against FBXL2 were subjected to **g** Western blot analyses, **h** MTS assays, or **i** colony formation assays. Three independent experiments were performed. H292 stable cells were subjected to **j** Western blot analyses or **k** xenograft tumor growth assays (*n* = 5/group). Photos of tumors and growth curves were presented. **p = 0.0063, ***p = 0.00025. H1975 cells stably expressing shRNAs against FBXL2 were subjected to **l** Western blot analyses, **m** MTS assays, or **n** Colony formation assays. Three independent experiments were performed. **o**−**r** Rosa26-LSL-EGFR^L858R/T790M^ mice were used to assess the effects of FBXL2 on EGFR^T790M^-mediated TKI resistance (*n* = 4 or 6/group). **o** Representative micro-CT images and photos of the lung were shown. Khaki, green, or purple-red represents heart, lung, or tumors, respectively. **p** The numbers of observable nodules in the lung surface were presented. Paraffin-embedded lungs were sectioned and subjected to either **q** H&E staining or **r** IHC analyses. Data were quantified by AOD. Data were presented as means ± SD (**a**, **c**, **h**−**i**, and **m**, **n**) or means ± SEM (**k**, **p**, and **r**). *p < 0.05, **p < 0.01, ***p < 0.001; by two-tailed Student’s *t*-test. Source data are provided as a Source data file.

Our results prompted us to verify the clinical relevance of FBXL2 and EGFR in NSCLC. As shown in Fig. 3a, analyses of TCGA database revealed that FBXL2 expression was significantly reduced in NSCLC, even in the cancer samples harboring EGFR gene mutations. In addition, analyses of tissue microarrays (TMA) showed that low levels of FBXL2 expression were observed in 66.67% (50 of 75) in human lung squamous cell carcinoma (LUSC) compared to adjacent tissues (Fig. 3b). Similarly, FBXL2 expression was reduced in lung tumor samples derived from Rosa26-LSL-EGFR^L858R/T790M^ mice (*n* = 8, Supplementary Fig. 3a). We further examined FBXL2 expression at various stages of human lung adenocarcinoma (LUAD). As shown in Fig. 3c, TMA analyses showed that FBXL2 was progressively reduced during lung adenocarcinoma development. Furthermore, a reverse correlation was observed between FBXL2 and EGFR protein expression in TMA of lung adenocarcinoma and lung squamous cell carcinoma (Fig. 3d, e and Supplementary Fig. 3b). Consistently, low expression of FBXL2 was reversely correlated with high expression of EGFR in NSCLC cell lines used in this study (Supplementary Fig. 3c). Moreover, analyses of Kaplan−Meier survival datasets showed that low expression of FBXL2 was associated with poor overall survival (OS) (Fig. 3f).

Our aforementioned results showed that FBXL2 is a key negative regulator of EGFR, we, therefore, investigated the effects of FBXL2 on cell proliferation and tumor growth using H292 cells, which express high levels of wild-type EGFR^31^. As shown in Fig. 3g−i and Supplementary Fig. 3d, silencing of FBXL2 led to upregulation of EGFR expression, AKT/ERK protein phosphorylation, and increased cell proliferation. Conversely, ectopic expression of wild-type FBXL2, but not FBXL2^C420S^, FBXL2^ΔF^, or FBXL2^4A^, markedly reduced EGFR protein levels, decreased AKT/ERK protein phosphorylation, and suppressed cell proliferation (Fig. 3j and Supplementary Fig. 3e). Furthermore, wild-type FBXL2, but not FBXL2^C420S^, significantly suppressed tumor growth in H292 xenograft mouse model (Fig. 3k). These results indicate that FBXL2 inhibits the growth of EGFR-overexpressed NSCLC.

The expression of EGFR^L858R/T790M^ mutant protein is frequently found in lung cancer patients, we then investigated the effects of FBXL2 on the growth of EGFR^L858R/T790M^-driven NSCLC in vitro and in vivo. As shown in Fig. 3l−n and Supplementary Fig. 4a, silencing of FBXL2 markedly upregulated EGFR^L858R/T790M^ expression, activated the EGFR downstream pathways, and promoted cell proliferation in H1975 cells. Conversely, ectopic expression of wild-type FBXL2, but not FBXL2^ΔF^ or FBXL2^4A^ mutant, reduced EGFR^L858R/T790M^ protein expression and inhibited the EGFR downstream pathways, concomitant with inhibition of cell proliferation and colony formation in H1975 cells (Supplementary Fig. 4b−d). To further substantiate the inhibitory role of FBXL2 on the growth of EGFR^L858R/T790M^-driven NSCLC in vivo, we generated a Cre-inducible Rosa26-LSL-EGFR^L858R/T790M^ lung tumor mouse model (Supplementary Fig. 4e). As shown in Fig. 3o−q, the Ade-Cre-induced expression of EGFR^L858R/T790M^ effectively drove lung tumor formation, which was significantly inhibited by lentiviral expression of HA-FBXL2, as evidenced by micro-CT scanning, measurement of lung tumor numbers, and H&E staining of tumor sections. Furthermore, lung lesions derived from HA-FBXL2-expressing mice exhibited a significant reduction of EGFR and p-EGFR expression (Fig. 3r and Supplementary Fig. 4f). Moreover, there were significantly reduced Ki67^+^ cells but no significant difference in cleaved caspase-3^+^ (CC3^+^) apoptotic cells (Supplementary Fig. 4f, g), suggesting that FBXL2 suppresses tumor growth primarily through inhibition of tumor cell proliferation. Together, these results indicate that FBXL2 can inhibit EGFR^L858R/T790M^-driven lung tumor growth.

### FBXL2 inhibits NSCLC growth via downregulation of EGFR expression

**Fig. 4:**
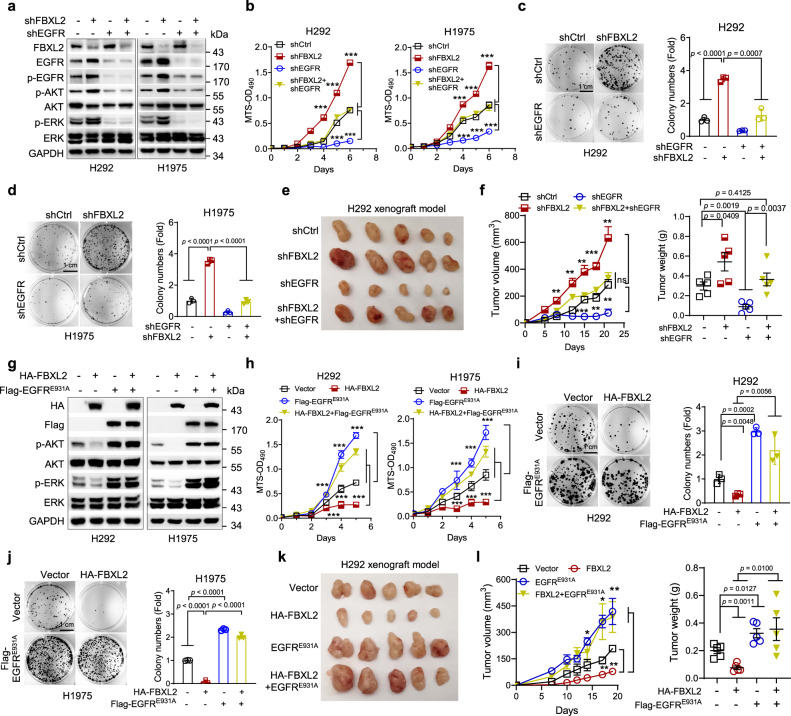
FBXL2 inhibits NSCLC growth via downregulation of EGFR expression. H292 or H1975 cells stably expressing either shFBXL2 or shEGFR, or both were subjected to **a** Western blot analyses, **b** MTS assays, or **c**, **d** colony formation assays. Three independent experiments were performed. Data were presented as means ± SD. **e**, **f** H292 cells stably expressing either shFBXL2 or shEGFR, or both were subjected to xenograft tumor growth assays (*n* = 5/group). Mice were euthanized by day 21 after inoculation and tumors were dissected and photographed. Tumor growth curves and tumor weight were presented as means ± SEM. H292 or H1975 cells stably expressing HA-FBXL2 or Flag-EGFR^E931A^, or both were subjected to **g** Western blot analyses, **h** MTS assays, or **i**, **j** colony formation assays. Three independent experiments were performed. Data were presented as means ± SD. **k**, **l** H292 cells stably expressing either HA-FBXL2 or Flag-EGFR^E931A^, or both were subjected to xenograft tumor growth assays (*n* = 5/group). Mice were euthanized by day 19 after inoculation and tumors were dissected and photographed. Tumor growth curves and tumor weight were presented as means ± SEM. *p < 0.05, **p < 0.01, ***p < 0.001; all by two-tailed Student’s *t*-test. Source data are provided as a Source data file.

To investigate whether downregulation of EGFR is responsible for FBXL2-induced inhibition of NSCLC growth, we performed the rescuing experiments. As shown in Fig. 4a−d, at the cellular level, simultaneous silencing of EGFR completely rescued FBXL2 knockdown-induced both activation of the EGFR downstream pathways and increased cell proliferation in H292 or H1975 cells. In addition, silencing of EGFR significantly rescued FBXL2 knockdown-induced tumor growth in H292 xenograft mouse model (Fig. 4e, f), indicating that silencing of FBXL2 promotes NSCLC cell proliferation and tumor growth via up-regulation of EGFR expression. To substantiate this conclusion, we further examined the effects of ectopic expression of EGFR^E931A^ on rescuing FBXL2-mediated inhibition of NSCLC growth. Our results showed that although EGFR^E931A^ was incapable of binding FBXL2, it retained wild-type EGFR kinase activity (Supplementary Fig. 5a). Ectopic expression of EGFR^E931A^ fully restored cell proliferation and tumor growth, both of which were suppressed by ectopic expression of FBXL2 (Fig. 4g−l). Together, these results indicate that FBXL2 suppresses cell proliferation and NSCLC growth via downregulation of EGFR expression. In keeping with this line, we examined the impacts of FBXL2 on growth of A549 and H1299 cells, both of which harbor a mutant Ras, a key downstream effector of the EGFR signaling. As shown in Supplementary Fig. 5b, c, while FBXL2 effectively inhibited EGFR expression, it had little effects on ERK activation or cell proliferation of A549 or H1299 cells, indicating that EGFR signaling is responsible for the effects of FBXL2 on NSCLC growth.

### Grp94 competes with FBXL2 for EGFR-binding and inhibition of Grp94 augments FBXL2-mediated suppression of TKI-resistant NSCLC growth

**Fig. 5:**
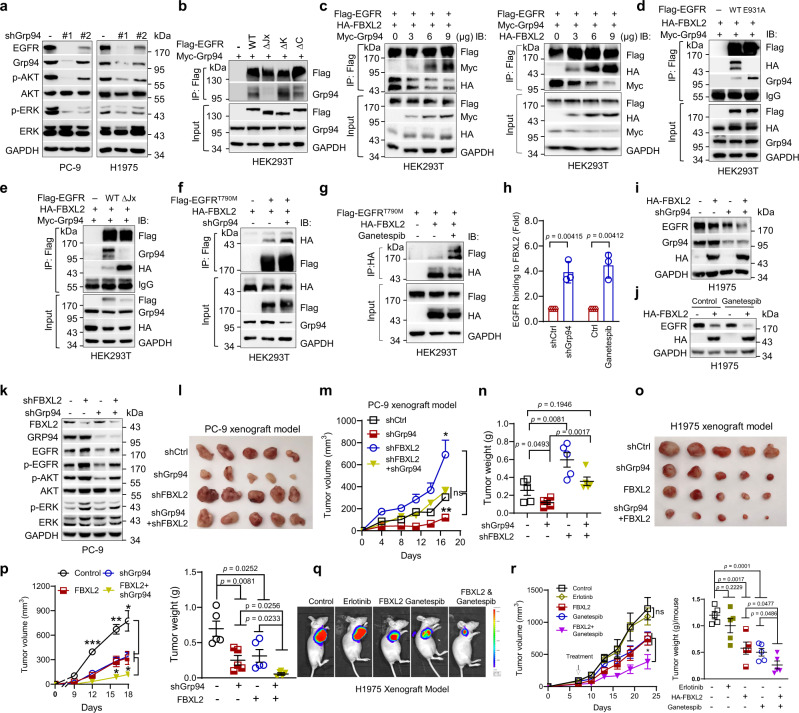
Grp94 competes with FBXL2 for EGFR binding, and inhibition of Grp94 promotes FBXL2-mediated EGFR degradation in the suppression of TKI-resistant lung tumor growth. **a** PC-9 or H1975 cells stably expressing shGrp94 were subjected to Western blot analyses. **b**, **c** HEK293T cells were co-transfected with indicated expressing plasmids for 36 h. Cells were then treated with MG132 for 4 h, followed by IP-Western analyses. **d**, **e** HEK293T cells were co-transfected with HA-FBXL2, Myc-Grp94, and indicated Flag-EGFR expressing plasmids for 36 h. Cells were then treated with MG132 for 4 h, followed by IP-Western analyses. HEK293T cells were co-transfected with Flag-EGFR^T790M^, HA-FBXL2, and either shCtrl or shGrp94 expressing plasmids overnight (**f**); or HEK293T cells were co-transfected with Flag-EGFR^T790M^ and HA-FBXL2 expressing plasmids overnight and were then treated with or without ganetespib (8 nM) for 12 h (**g**). Cells were treated with MG132 for 4 h prior to IP-Western analyses. The affinity of EGFR binding to FBXL2 was quantified and presented as means ± SD. **i** H1975 cells stably expressing HA-FBXL2 or shGrp94, or both were subjected to Western blot analyses. **j** H1975 cells stably expressing HA-FBXL2 were treated with or without ganetespib (8 nM) for 24 h prior to Western blot analyses. PC-9 cells stably expressing either shFBXL2 or shGrp94, or both were subjected to **k** Western blot analyses or **l**−**n** xenograft tumor growth assays (*n* = 5/group). Photos of tumors, growth curve, and tumor weight were presented. **o**, **p** H1975 cells stably expressing either HA-FBXL2 or shGrp94, or both were subjected to xenograft tumor growth assays (*n* = 5/group). Photos of tumors, growth curve, and tumor weight were presented. **q**, **r** H1975 cells (5 × 10^5^) stably expressing HA-FBXL2 or vector were subjected to xenograft tumor growth assays (*n* = 5/group). Mice were treated with erlotinib or ganetespib. Representative bioluminescence imaging, growth curve and tumor weight were presented. The experiment was repeated three times independently with similar results (**a**−**g** and **i**−**k**). Data were presented as means ± SEM (**m**, **n**, **p**, and **r**). *p < 0.05, **p < 0.01, ***p < 0.001; all by two-tailed Student’s *t*-test. Source data are provided as a Source data file.

Since FBXL2 is also colocalized with EGFR on endoplasmic reticulum, we reasoned that endoplasmic reticulum may be involved in FBXL2-mediated regulation of EGFR protein stability. We, therefore, investigated whether Grp94, an ER-resident member of Hsp90 family critically involved in protein homeostasis^32^, plays a role in the regulation of EGFR protein stability. As shown in Fig. 5a, silencing of Grp94 markedly downregulated EGFR expression in PC-9 or H1975 cells. Grp94 was able to bind the juxtamembrane (Jx) domain of EGFR, but not FBXL2 (Fig. 5b and Supplementary Fig. 6a). Notably, Grp94 interfered FBXL2 binding to EGFR in a dose-dependent manner. Conversely, FBXL2 interfered Grp94 binding to EGFR in a dose-dependent manner (Fig. 5c). These results suggest that FBXL2 and Grp94 compete each other for EGFR binding. Consistently, EGFR^E931A^, a mutant unable to interact with FBXL2, bound more Grp94, whereas EGFR^∆Jx^, a mutant defective in interaction with Grp94, bound more FBXL2 (Fig. 5d, e). Furthermore, either silencing of Grp94 or inhibition of Grp94 by the small molecule inhibitor, ganetespib, significantly enhanced interaction between FBXL2 and EGFR^T790M^, leading to accelerated FBXL2-mediated degradation of EGFR^L858R/T790M^ protein (Fig. 5f−j). Moreover, clinical analyses of TCGA database revealed low expression of FBXL2 and high expression of Grp94 in lung adenocarcinoma (LUAD) or lung squamous cell carcinoma (LUSC) (Supplementary Fig. 6b, c).

We next investigated whether the effects of Grp94 on cell proliferation and NSCLC tumor growth is dependent on FBXL2. As shown in Supplementary Fig. 6d, silencing of Grp94 inhibited both EGFR expression and proliferation of H1975 cells, which was well rescued by ectopic expression of EGFR^T790M^. Simultaneous silencing of FBXL2 could also well rescue Grp94 knockdown-induced inhibition of EGFR and tumor growth in PC-9 xenograft mouse model, suggesting that Grp94 impacts EGFR expression and tumor growth via FBXL2 (Fig. 5k−n). Conversely, ectopic expression of Grp94 significantly restored expression of EGFR and PC-9 xenograft tumor growth, both of which were suppressed by ectopic expression FBXL2 (Supplementary Fig. 6e−h). Moreover, either ectopic expression of Grp94 or silencing of FBXL2 could also well rescue ganetespib-induced inhibition of EGFR and cell proliferation (Supplementary Fig. 6i, j), indicating that the effects of ganetespib on inhibition of EGFR expression and cell proliferation is dependent on Grp94 and FBXL2.

We next investigated the effects of combinatory targeting Grp94 and FBXL2 on xenograft lung tumor growth. As shown in Fig. 5o, p silencing of Grp94 significantly augmented FBXL2-mediated suppression of H1975 xenograft tumor growth. Consistently, ganetespib could also significantly augment FBXL2-mediated suppression of cell proliferation and H1975 xenograft tumor growth (Fig. 5q, r and Supplementary Fig. 7a), concomitant with reduction of total and phosphorylated EGFR protein levels and reduced Ki67^+^ cells (Supplementary Fig. 7b−d). Moreover, ganetespib significantly increased apoptotic cells (CC3^+^) (Supplementary Fig. 7d). Therefore, inhibition of Grp94 by ganetespib augments FBXL2-medicated inhibition of TKI-resistant lung tumor growth via suppressing cell proliferation and promoting apoptotic cell death.

### Nebivolol is an activator of FBXL2 in facilitating EGFR degradation and inhibiting NSCLC growth

**Fig. 6:**
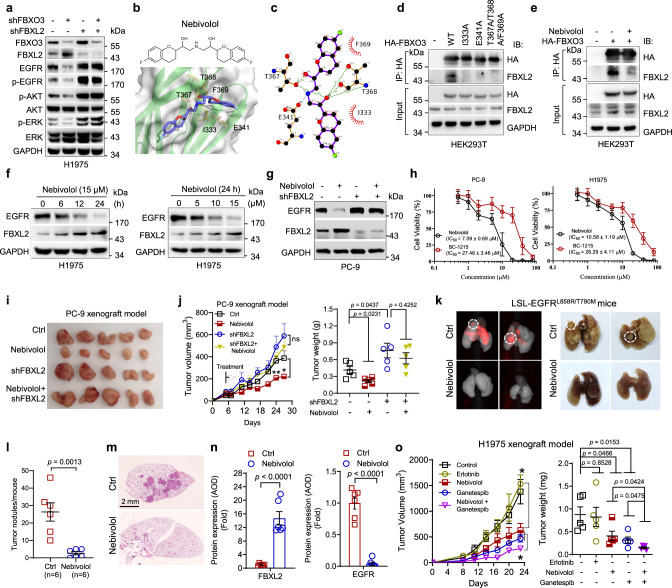
Nebivolol upregulates FBXL2 to facilitate EGFR degradation and inhibit tumor growth. **a** H1975 stable cells were subjected to Western blot analyses. **b** The chemical structure of nebivolol and a molecular docking model of nebivolol and FBXO3-ApaG domain. **c** A schematic diagram of the projected interaction by LigPlot^65^ between indicated residues in FBXO3-ApaG domain and nebivolol. The hydrogen bonds or electrostatic interaction are highlighted in green or blue dash lines, respectively. The hydrophobic interaction is remarked as the short radiating red lines. HEK293T cells transfected with the indicated expressing plasmids were treated without (**d**) or with (**e**) 10 µM nebivolol for 36 h. Cells were treated with MG132 prior to IP-Western analyses. **f** H1975 cells were treated with nebivolol for an indicated dose and time interval prior to Western blot analyses. **g** PC-9 stable cells were treated with 10 µM nebivolol for 48 h prior to Western blot analyses. **h** The IC_50_ of PC-9 or H1975 cells treated with nebivolol or BC-1215 were determined by MTS assays. Data were presented as means ± SD. **i**, **j** PC-9 stable cells subjected to xenograft tumor growth assays (*n* = 5/group). Photos of tumors, growth curve, and tumor weight were presented. **k**−**n** Transgenic mice (Rosa26-LSL-EGFR^L858R/T790M^) were used (*n* = 5/group). **k** The representative fluorescent images and photos of lungs were shown. Fluorescence in red indicates high EGFR expression. Representative tumors with high levels of EGFR expression were shown in broken line of circles. **l** The numbers of observable nodules on the lung surfaces were presented. Paraffin-embedded lungs were sectioned and subjected to either **m** H&E staining or **n** IHC analyses. A specific polyclonal antibody for mouse FBXL2 was custom-made from Hangzhou HuaAn Biotechnology Co., Ltd. (Hangzhou, China). **o** H1975 cells (5 × 10^5^) were subjected to xenograft tumor growth assays (*n* = 5/group). Growth curve and tumor weight were presented. The experiment was repeated three times independently with similar results (**a** and **d**−**h**). Data were presented as means ± SEM (**j**, **n**, and **o**). *p < 0.05, **p < 0.01, ***p < 0.001; by two-tailed Student’s *t*-test. Source data are provided as a Source data file.

Given the important role of FBXL2-mediated EGFR^T790M^ degradation in suppression of TKI-resistant NSCLC growth, we reasoned that targeting FBXL2 could be beneficial in the treatment of TKI-resistant NSCLC. To preclinically prove this concept, we aimed to identify activators of FBXL2. It has been reported that FBXL2 is targeted and degraded by the E3 ubiquitin ligase FBXO3 and that the FBXO3-ApaG domain is required for FBXL2 interaction^26,33^. Therefore, we screened chemical libraries for small molecule inhibitors that can bind to the FBXO3-ApaG domain to interfere FBXO3−FBXL2 interaction, resulting in FBXL2 protein stabilization and thereby facilitating EGFR protein degradation. With this regard, we first examined whether inhibition of FBXO3 could lead to a reduction of EGFR expression. Indeed, silencing of FBXO3 led to an increase in FBXL2 protein levels, reduced EGFR^L858R/T790M^ expression, concomitant with inhibited EGFR downstream signaling in H1975 cells, all of which were well rescued by simultaneous silencing of FBXL2 (Fig. 6a and Supplementary Fig. 8a).

By virtual screening of the DrugBank database consisting of 2,373 FDA-approved drugs^34^, the small chemical compounds capable of binding to FBXO3-ApaG domain were scored (Supplementary Fig. 8b). Three top hits, nebivolol, flibanserin, and raltegravir, were obtained (Supplementary Fig. 8c and Fig. 6b). Notably, nebivolol, a beta-blocker for the treatment of patients with high blood pressure or heart failure^35^, was identified as the best hit as evidenced by the initial bioassay (Supplementary Fig. 8d). The docking results projected that nebivolol could fit in a dumbbell-shaped cavity in the ApaG domain. There are five amino acid residues (I331, E341, T367, T368, and F369) in the cavity center. T367 and T368 were projected to form hydrogen bonds with nebivolol, while E341 was in close proximity to the positively charged amines of nebivolol, suggesting a strong charge−charge electrostatic interaction between the groups. The heterocyclic groups at two wings of nebivolol were predicted to fit in the shape-complementary cavity with hydrophobic interactions between them (Fig. 6b, c). In keeping with this model, the FBXO3 proteins with these five amino acids mutated either alone or in combination showed much reduced binding affinity with endogenous FBXL2 (Fig. 6d). Taken together, these results suggest that nebivolol is a potential small molecule that could disrupt the FBXO3−FBXL2 interaction.

We then examined whether nebivolol could interfere FBXO3−FBXL2 interaction and inhibit EGFR expression. As shown in Fig. 6e, nebivolol was able to interfere FBXL2 binding to FBXO3. Furthermore, nebivolol markedly upregulated FBXL2 and downregulated EGFR expression in both time- and dose-dependent manners in PC-9 or H1975 cells (Fig. 6f and Supplementary Fig. 8e). Notably, nebivolol was unable either to increase FBXL2 expression or to reduce EGFR protein levels upon silencing of FBXO3 (Supplementary Fig. 8f). In addition, nebivolol could not inhibit EGFR expression upon silencing of FBXL2 (Fig. 6g), indicating that the effect of nebivolol on EGFR expression is FBXO3/FBXL2-dependent. Consistent with this notion, nebivolol-induced EGFR reduction was rescued by MG132 but not by chloroquine, in a manner similar to ectopically expressed FBXL2 (Supplementary Fig. 8g). These results indicate that nebivolol can disrupt FBXO3−FBXL2 interaction, resulting in upregulation of FBXL2 and facilitation of EGFR degradation.

Furthermore, nebivolol exhibited strong inhibitory effects on the viability of NSCLC cells in a dose-dependent manner (Fig. 6h). BC-1215, a known inhibitor of FBXO3−FBXL2 interaction^26^, was used in parallel. Notably, while nebivolol significantly inhibited cell proliferation in PC-9 cells bearing wild-type Ras, it exhibited little effects in A549 cells that possess K-Ras^G12S^ (Supplementary Fig. 8h), indicating that EGFR signaling is mainly responsible for the inhibitory effects of nebivolol on cell proliferation of NSCLC. Moreover, nebivolol significantly inhibited cell proliferation and tumor growth in PC-9 xenograft mouse model, while it failed to do so upon silencing of FBXL2 (Supplementary Fig. 8i and Fig. 6i, j). These results indicate that upregulation of FBXL2 by nebivolol is responsible for its effects on the growth inhibition.

We next evaluated the therapeutic potential of nebivolol using the EGFR^L858R/T790M^-driven NSCLC mouse model. As shown in Fig. 6k−m, nebivolol strongly inhibited EGFR expression examined by Pearl Trilogy Imagers and suppressed EGFR^L858R/T790M^-driven lung tumor formation, as evidenced by a significant reduction of the lung tumor numbers and tumor size. Furthermore, IHC analyses of the lung lesions derived from nebivolol-treated mice showed significant upregulation of FBXL2 expression and dramatically reduced expression of EGFR as well as cyclin D3, a known substrate of FBXL2^25^, compared to that of untreated mice (Fig. 6n and Supplementary Fig. 9a, b). Notably, nebivolol treatment led to much reduced Ki67^+^ cells, with no significant alteration in cleaved caspase-3^+^ (CC3^+^) apoptotic cells (Supplementary Fig. 9b). Furthermore, inhibition of Grp94 by ganetespib significantly augmented nebivolol-mediated both reduction of EGFR^L858R/T790M^ expression and inhibition of tumor growth (Fig. 6o and Supplementary Fig. 9c−h). Together, these results demonstrate that nebivolol is a FBXL2 activator that acts to inhibit TKI-resistant tumor growth, which is augmented by ganetespib.

### Activation of FBXL2 overcomes osimertinib resistance of NSCLC

**Fig. 7:**
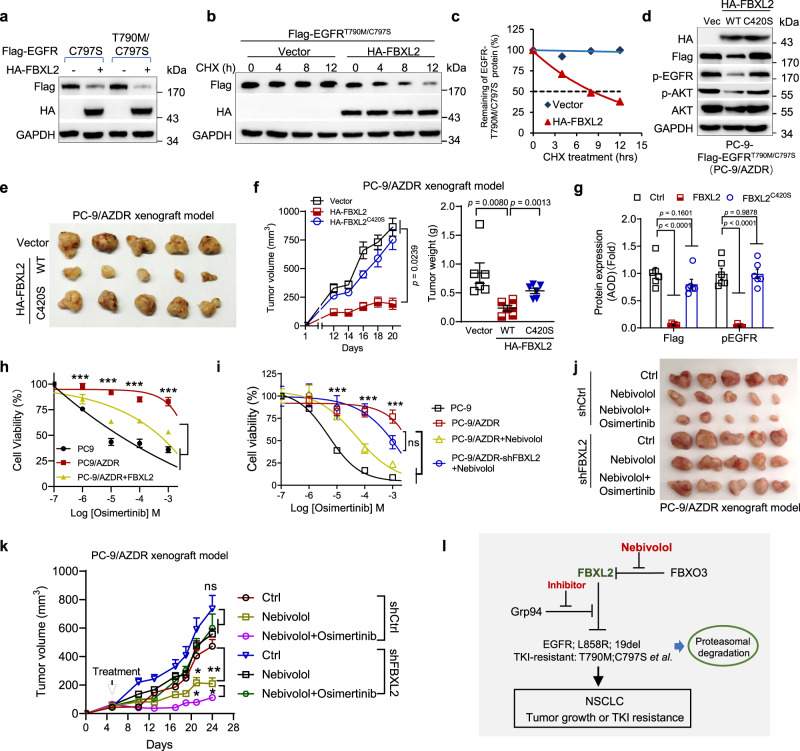
Activation of FBXL2 overcomes osimertinib resistance of NSCLC. **a** HEK293T cells were co-transfected with indicated expressing plasmids for 36 h, followed by Western blot analyses. Data are representative immunoblots of three independent assays. **b**, **c** HEK293T cells were co-transfected with Flag-EGFR^T790M/C797S^ in the presence of HA-FBXL2 or a vector control for 36 h, and were then treated with 50 µg/mL cycloheximide (CHX) for an indicated time interval prior to Western blot analyses. The EGFR protein levels were quantified and a plot representing protein half-life was presented. PC-9-Flag-EGFR^T790M/C797S^ cells stably expressing HA-FBXL2 or HA-FBXL2^C420S^ were subjected to **d** Western blot analyses or **e**−**g** xenograft tumor growth assays (*n* = 6/group). Photos of tumor, growth curve, and tumor weight were presented. Paraffin-embedded tumors were subjected to IHC analyses. Data were quantified by AOD. Data were presented as means ± SEM. **h** PC-9 cells stably expressing EGFR^T790M/C797S^ (PC-9/AZDR) were infected with lentivirus encoding HA-FBXL2. Cells were treated with an indicated dose of osimertinib for 72 h prior to MTS analyses. Data derived from three independent experiments in triplicates were presented as means ± SD. ***p < 0.001. **i** PC-9/AZDR cells stably expressing shFBXL2 were treated with an indicated dose of osimertinib in the absence or presence of 5 μM nebivolol for 48 h followed by MTS assays. Data from three independent experiments in triplicates were presented as means ± SD. **j**, **k** PC-9/AZDR stable cells (2 × 10^5^) were subjected to xenograft tumor growth assays (*n* = 5/group). Mice were administrated with nebivolol alone or in combination with osimertinib. Photos of tumors and tumor growth curves were shown. Data were presented as means ± SEM. **l** A working model. The E3 ubiquitin ligase FBXL2 targets EGFR and EGFR mutants for proteasomal degradation, resulting in inhibition of tumor growth and TKI resistance of NSCLC. Grp94 binds to and protects EGFR from FBXL2-mediated degradation. The FDA-approved drug nebivolol can disrupt FBXO3−FBXL2 interaction to stabilize FBXL2 and degrade EGFR, thereby inhibiting both NSCLC growth and TKI resistance.*p < 0.05, **p < 0.01, ***p < 0.001; all by two-tailed Student’s *t*-test. Source data are provided as a Source data file.

Resistance to EGFR-TKIs is a major hurdle for NSCLC treatment, and currently, there is no approved new generation of TKIs for osimertinib-resistant NSCLC. Thus, we investigated the effects of FBXL2 on osimertinib resistance of NSCLC. To this end, we first examined the effects of FBXL2 on the expression of EGFR mutant proteins that are resistant to osimertinib. As shown in Supplementary Fig. 10a, FBXL2 markedly inhibited expression of EGFR L792H, G796D, C797S, and L718Q mutant proteins, all of which are shown to mediate resistance to osimertinib^14,15,36,37^. In addition, FBXL2 also markedly suppressed expression of EGFR E709K, L798I, and L844V, all of which were identified from NSCLC patients resistance to WZ4002 and CO1686, two potential 3rd-generation TKIs in preclinical studies^38,39^ (Supplementary Fig. 10a). In particular, FBXL2 inhibited expression and shortened the half-life of EGFR^T790M/C797S^ protein, which confers resistance to all currently available EGFR-TKIs^40^ (Fig. 7a−c). Moreover, FBXL2 was able to bind EGFR^C797S^ or EGFR^T790M/C797S^ protein (Supplementary Fig. 10b). These results indicate that FBXL2 can inhibit the expression of EGFR mutant proteins that are resistant to osimertinib.

To further investigate the effects of activation of FBXL2 on the growth of osimertinib-resistant NSCLC, we established PC-9 cells stably expressing EGFR^T790M/C797S^ (PC-9/AZDR), which are resistant to osimertinib (Supplementary Fig. 10c). As shown in Fig. 7d and Supplementary Fig. 10d, e, nebivolol treatment or ectopic expression of FBXL2 reduced EGFR^T790M/C797S^ protein expression, concomitant with suppression of cell proliferation. In addition, nebivolol-induced suppression of cell proliferation was markedly enhanced by Grp94-specific inhibitor (Grp94 inhibitor-1, iGrp94-1^41^) (Supplementary Fig. 10f). Furthermore, wild-type FBXL2, but not FBXL2^C420S^, dramatically inhibited PC-9/AZDR xenograft tumor growth, concomitant with significantly reduced EGFR expression and Ki67^+^ cells (Fig. 7e−g and Supplementary Fig. 10g). These results demonstrate that activation of FBXL2 can effectively inhibit osimertinib-resistant NSCLC growth.

We then investigated the effects of FBXL2 together with osimertinib on TKI-resistant NSCLC growth in vitro and in vivo. IC_50_ assay showed that either ectopic FBXL2 expression or nebivolol markedly reduced resistance to erlotinib, gefitinib, or osimertinib (Fig. 7h, i and Supplementary Fig. 11a, b). In addition, the combination of nebivolol and osimertinib dramatically inhibited osimertinib-resistant NSCLC growth in PC-9/AZDR xenograft mouse model (Fig. 7j, k), with no observable liver injury or alteration of body weights (Supplementary Fig. 11c). Notably, nebivolol was unable to reduce osimertinib resistance upon FBXL2 silencing (Fig. 7i−k). In agreement with this observation, either ectopic FBXL2 expression or nebivolol had little effects on EGFR^E931G^ mutant-induced erlotinib or osimertinib resistance (Supplementary Fig. 11d, e). Together, these results demonstrate that a combination of nebivolol and osimertinib can effectively overcome EGFR^T790M/C797S^-induced osimertinib resistance of NSCLC in a FBXL2-dependent.

## Discussion

Lung cancer is the leading cause of cancer-related deaths worldwide. The major NSCLC therapeutic regime based on EGFR-TKI has been evolved from gefitinib (2003), erlotinib (2004), afatinib (2013) to osimertinib (2015), all of which have greatly improved survival outcomes and prognosis for patients. However, nearly all patients eventually develop acquired drug resistance following treatment with these EGFR-TKIs. Along with gene amplification of *MET* or *HER2* as well as mutations in *Ras*, *BRAF*, or *PIK3CA*, EGFR^T790M^ is the most common mutation resistant to 1st- and 2nd-generation EGFR-TKIs (gefitinib, erlotinib, and afatinib)^42^. The 3rd-generation EGFR-TKI, osimertinib, has been developed to overcome EGFR^T790M^-mediated TKI resistance and used as a first-line therapy for EGFR-mutated NSCLC. Unfortunately, new mutations resistant to osimertinib, exemplified by EGFR^C797S^, have been rapidly emerged as early as in 2015^14^. Therefore, it is of great importance to develop new strategies to overcome EGFR-TKI resistance.

In this study, we have presented preclinical evidence, in proof of concept, that targeting FBXL2/Grp94 to facilitate EGFR protein turnover may be a new therapeutic strategy for the treatment of TKI-resistant NSCLC. We demonstrate that FBXL2 is an E3 ubiquitin ligase targeting wild-type EGFR, EGFR activating mutants (L858R or 19del), and EGFR TKI-resistant mutants (including EGFR T790M or T790M/C797S) for proteasomal degradation, resulting in inhibition of both tumor growth and TKI resistance in vitro and in vivo. Clinical analyses have also established the connection between FBXL2 and EGFR such that expression of FBXL2 is reversely correlated with EGFR in NSCLC and that FBXL2 expression is reduced in NSCLC and is associated with poor clinical prognosis. Importantly, we have identified that nebivolol, an FDA-approved drug^35^, is an activator of FBXL2. Nebivolol exhibits strong inhibitory effects on osimertinib-resistant NSCLC growth. Combination of nebivolol and osimertinib effectively overcomes osimertinib resistance in vivo. Our results show that nebivolol can interfere FBXL2 interaction with FBXO3 in vivo. In order to validate that nebivolol directly interacts with FBXO3-ApaG, we aimed to perform in vitro binding assays using recombinant FBXO3-ApaG protein, which were unsuccessful due to the obstacles of obtaining purified recombinant FBXO3-ApaG protein attributed to its insolubility. This issue deserves further investigation.

The biological function of FBXL2 seems to be complex with regard to cancer development. While several studies show that FBXL2 can promote the degradation of free p85β or IP3R3 to inhibit autophagy and apoptosis^27,28^, FBXL2 can also target several oncoproteins, including cyclin D2, cyclin D3, or Aurora B for proteasomal degradation, and consequently inhibit cell proliferation and tumor growth^24,25,43^. Our results strongly support the notion that FBXL2 is a tumor suppressor through targeting and promoting EGFR degradation, consequently leading to inhibition of EGFR downstream signaling and suppression of tumor growth. Importantly, our results show that inhibitory effects of FBXL2 on cell proliferation and NSCLC growth are mainly dependent on EGFR signaling. Firstly, FBXL2-mediated inhibition of cell proliferation and tumor growth can be completely rescued by restoration of EGFR expression. Secondly, FBXL2 inhibits cell proliferation in H292, PC9 or H1975 cells, all of which bear wild-type Ras alleles, while it exhibits little effects on A549 or H1299 cells harboring activated Ras, an essential downstream effector of EGFR, in keeping with the clinical guideline that EGFR-TKI-based treatment of NSCLC is not applicable to patients with activated Ras^44,45^. Furthermore, either FBXL2 or nebivolol can effectively overcome erlotinib or osimertinib resistance mediated by EGFR^T790M^ or EGFR^C797S^, but not mediated by FBXL2-insensitive EGFR^E931G^. Together, these results provide strong evidence that FBXL2-mediated suppression of NSCLC growth and TKI resistance is dependent on FBXL2-induced EGFR protein degradation. However, this study does not exclude the role of FBXL2-mediated regulation on other signaling molecules, such as cyclin D3, in NSCLC growth. Notably, it has been reported that vinorelbine, a clinically used chemotherapy drug, can target tubulin to sensitize EGFR TKI-resistant cells^46^. Vinorelbine is shown to increase FBXL2 expression and downregulate cyclin D3 expression to induce cell apoptosis^25^. Therefore, whether vinorelbine-mediated FBXL2 upregulation plays a role in EGFR destabilization deserves further investigation. In addition, it would be interesting to explore whether the combination of vinorelbine and nebivolol can better inhibit tumor growth and TKI resistance.

EGFR protein homeostasis is critically important in a variety of different biological processes, disruption of which can lead to various human diseases^47^. In this study, we show that EGFR protein homeostasis is maintained by both ligand-dependent and ligand-independent manners involved in lysosomal and proteasome degradation pathways. It has been shown that EGFR expression is tightly regulated upon EGF stimulation. The E3 ubiquitin ligase c-Cbl can interact with and promote EGFR degradation via lysosome in response to EGF^48^. Of note, the deubiquitinating enzyme (DUB) USP2a and UCHL1 can deubiquitinate and stabilize EGFR protein^49,50^. USP9X, on the other hand, can regulate EGFR protein internalization and trafficking thereby affecting EGFR protein turnover^5^. Our results show that FBXL2 is a critical E3 ubiquitin ligase promoting EGFR proteasomal degradation independent of EGF stimulation. Under physiological circumstances, c-Cbl promotes EGFR protein lysosomal degradation upon stimulation of EGF, thus providing a pivotal mechanism to attenuate plasma membrane-associated EGFR signaling^48^. To maintain proper protein homeostasis, the newly synthesized EGFR protein levels in ER must be taken into consideration. Protein stability of newly synthesized EGFR can be controlled by CHIP/Hsp70 and Hsp90^51–53^. In this study, we found that Grp94 can compete with FBXL2 to protect EGFR from FBXL2-mediated degradation. Since FBXL2 and Grp94 bind to different segments in EGFR, it is plausible that binging of Grp94 to EGFR may alter the protein confirmation thus preventing FBXL2 for stable interaction with EGFR, and vice versa. Therefore, EGFR protein stability is controlled at multiple levels of complex regulation, including specific E3 ligases, DUBs, proteases, and components of proteasome and lysosomes, as well as the modifying factors affecting the expression and activities of these enzymes. In this study, we demonstrate that FBXL2 is a critical E3 ubiquitin ligase for EGFR since alteration of FBXL2 expression profoundly impacts EGFR protein stability.

Notably, emerging evidence indicates that selective inhibition of Grp94 may represent an effective approach for cancer treatment^18,54^. In this study, we show that ganetespib suppresses NSCLC cell proliferation through inhibition of Grp94 and that combination of FBXL2 activation and Grp94 inhibition exhibits strong growth suppression effects on EGFR-mutated NSCLC. Together, targeting FBXL2, as exemplified by nebivolol, in combination with EGFR-TKI or Grp94-specific inhibitor, may represent a putative strategy for EGFR-targeted therapy of NSCLC, especially osimertinib-resistant NSCLC.

## Methods

### Cell culture and drug treatment

H1299 and HEK 293T cells were grown in DMEM (Gibco, NY, USA) containing 10% FBS supplemented with penicillin (100 U/mL)/streptomycin (100 μg/mL). Human NSCLC cells H292, PC-9 or H1975 were grown in RPMI-1640 containing 10% FBS supplemented with penicillin (100 U/mL)/streptomycin (100 μg/mL) (Gibco, NY, USA). H1299 and H292 cells contain wild-type EGFR, H1975 or PC-9 cells bear EGFR^L858R/T790M^ or exon 19 deletion mutation, respectively. Cells were cultured at 37 °C in a humidified 5% CO_2_ incubator.

MG132 (S2619), erlotinib (S1023), gefitinib (S1025), ganetespib (STA-9090) (S1159), nebivolol HCl (S1549), flibanserin (S3716), raltegravir (S2005), and osimertinib (AZD9291) (S7297) were purchased from Selleck Chemicals (Houston, USA). BC-1215 (SML1049), chloroquine diphosphate salt (C6628), and cycloheximide (C7698) were purchase from Sigma-Aldrich (St. Louis, USA). Recombinant Human EGF (236-EG) was purchase from R&D systems (Minnesota, USA).

### Plasmids and lentiviral infection

The shRNA library for human E3 ubiquitin ligases (TRC library, RHS4896) used in this study was purchased from Thermo Scientific Open Biosystems (Massachusetts, USA). The short hairpin RNAs (shRNAs) targeting human FBXL2, FBXO3, EGFR, or Grp94 were generated by the insertion of specific oligos into a pLKO.1-puromycin lentiviral vector. A pLVX-puro vector was used to generate recombinant lentiviruses expressing either human FBXL2, FBXL2^4A^, FBXL2^ΔF^, FBXL2^C420S^, wild-type EGFR, EGFR^L858R^, EGFR^T790M^, EGFR^E709K^, EGFR^L792H^, EGFR^L798I^, EGFR^G796D^, EGFR^C797S^, EGFR^L718Q^, EGFR^L844V^, EGFR^L692V^, EGFR^T790M/C797S^, EGFR^K806A/D807A^, EGFR^S720A^, EGFR^K875A^, EGFR^S921A/E922A^, EGFR^E931A^, EGFR^E931G^, or Grp94. All the constructs including mutants generated by KOD-Plus-Mutagenesis kit (SMK-101, Toyobo Osaka) were confirmed by direct DNA sequencing. All primers used in this study were listed in Supplementary Table 1. Recombinant lentiviruses were amplified in HEK293T cells.

### Western blot analysis, co-immunoprecipitation assay, and immunofluorescence staining

For Western blot analyses, cells were washed twice with PBS and lysed with EBC250 buffer (250 mM NaCl, 25 mM Tris-HCl, pH 7.4, 0.5% Nonidet P-40 and 50 mM NaF) supplemented with a complete Protease inhibitor cocktail. Equal amounts of total protein were fractionated by SDS/PAGE, transferred into PVDF membrane. Membranes were blocked in 4% non-fat dry milk and incubated with primary antibody and HRP-conjugated secondary antibody for subsequent detection by chemiluminescence (Bio-Rad). Gel and blot images were analyzed using Image Lab Software 3.0. The uncropped and unprocessed gel or blot figures are provided as a Source Data file.

For endogenous Co-IP, cells were lysed with 0.25% NP-40 lysis buffer (20 mM Tris-HCl, 125 mM NaCl, 5 mM MgCl_2_, 0.2 mM EDTA, 12% Glycerol, and 0.25% Nonidet P-40). Equal amounts of total protein were incubated with primary FBXL2 antibodies or normal Rabbit IgG overnight for 7 h at 4°C, and then 30 µl of protein A beads were added for an additional 2 h of incubation. For exogenous Co-IP, anti-HA beads (or anti-flag beads) were added to equal amounts of total protein and incubated overnight. Beads were centrifuged (500 g for 30 s) and washed three times using wash buffer (20 mM Tris-HCl, 125 mM NaCl, 5 mM MgCl_2_, 0.2 mM EDTA, and 0.1% Nonidet P-40). The beads were heated at 100 °C for 10 min before SDS-PAGE and immunoblotting. Anti-FLAG M2 affinity gel (A2220) was purchased from Sigma-Aldrich (St. Louis, USA). Pierce Anti-HA magnetic beads (#88836) were purchased from Thermo Fisher Scientific (Waltham, MA, USA).

For immunofluorescence staining, cells in 24-well culture slides were fixed with 4% paraformaldehyde for 15 min, permeabilized with 0.1% Triton-100 for 15 min., blocked with 5% BSA for 1 h, and stained with specific primary antibodies followed by corresponding secondary antibodies. Nuclei were counterstained with DAPI. Images were captured using a confocal fluorescent microscope. The EGFR on the plasma membrane and ER were quantified by LAS_X software.

The following antibodies were used in Western blot analysis and co-immunoprecipitation assay: antibodies specific either for EGFR (#4267, 1:1000), p-EGFR (#3777, 1:1000), ERK (#9102, 1:1000), phospho-ERK (#9101, 1:1000), AKT(#9272, 1:1000), p-AKT(#4058, 1:1000), Grp94 (#20292, 1:1000), Na/K ATPase (#3010, 1:1000), LC3B (#2775, 1:1000), HA (#3724, 1:1000) or p21 (#2947, 1:1000) were purchased from Cell Signaling Technology (MA, USA). Antibodies specific for Grp78 antibody (ab-21685, 1:1000) or FBXL2 (ab-153842, 1:1000) were purchased from Abcam (Cambridge, UK). Antibody specific for c-Myc (sc-40, 1:100), Flag (F1804, 1:1000), or TRAF3 (D160776, 1:1000) was purchased from Santa Cruz Biotechnology (CA, USA), Sigma-Aldrich (St. Louis, USA), or Sangon Biotech (Shanghai, China), respectively. Goat anti-mouse IgG-HRP (sc-2005, 1:3000) or goat anti-rabbit IgG-HRP (sc-2004, 1:3000) antibodies was purchased from Santa Cruz Biotechnology (CA, USA). The following antibodies were used in immunofluorescence staining: antibodies specific for EGFR (Cell Signaling Technology, CST-4267, 1:100), Flag (Sigma-Aldrich, F1804, 1:50), or Grp78 (Abcam, ab-21685, 1:200). Rhodamine (TRITC)–conjugated AffiniPure Donkey Anti-mouse IgG (#715-025-150, 1:160) or anti-Rabbit IgG (#711-025-152, 1:160) and Fluorescein (FITC)-conjugated AffiniPure Donkey Anti-mouse IgG (#715-095-150, 1:160) or Anti-Rabbit IgG (#711-095-152, 1:160) were purchased from Jackson Immuno Research (PA, USA). In addition, APC anti-human EGFR antibody (352905, 5 µ/Test) and APC Mouse IgG1, κ Isotype Ctrl (FC) antibody (400121, 5 µ/Test) were purchased from Biolegend (CA, USA).

### Fractionation of membrane and cytosol proteins

Minute Plasma Membrane Protein Isolation and Cell Factionation Kit (SM-005, Invent Biotechnologies, Plymouth, USA) were used to fractionate membrane and cytosol proteins according to the manufacturer’s instructions.

### Cell proliferation analyses

Cell proliferation was determined by MTS assay. Briefly, cells were plated at a density of 500−3000 cells per well in 96-well microplates and grown overnight. Cells were treated with indicated inhibitors and then incubated with 20 μL MTS (G3582, Promega, Fitchburg, USA) for 1 h at 37 °C prior to measuring absorbance at OD = 490 nm.

### In vivo and in vitro ubiquitination assays

For in vivo ubiquitination assay, HEK293T cells were co-transfected with expressing plasmids encoding Flag-EGFR, FBXL2, and either wild-type HA-ubiquitin, HA-ubiquitin-Lys 48-only or HA-ubiquitin-Lys 63-only. Cells were grown overnight and were then treated with 20 μM MG132 for 4 h before harvesting. Cell lysates were immunoprecipitated using anti-Flag resin, followed by Western blot analyses.

In vitro ubiquitination assay was performed as described^28^. Briefly, HEK 293T cells were co-transfected with Flag-EGFR and HA-FBXL2 or HA-FBXL2^∆F^ expressing plasmids. Thirty-six hours post transfection, cells were treated with 20 μM MG132 for 4 h before immunoprecipitation with anti-HA beads, which were then added to the in vitro ubiquitylation mixture containing 0.1 μM E1 (UBE1, 23-021, Merck Millipore), 0.25 μM Ubch3 (23-022, Merck Millipore), 0.25 μM Ubch5c (23-035, Merck Millipore), 2 mM ATP (FLAAS, Sigma-Aldrich) in the presence or absence of 1 μM ubiquitin aldehyde (Millipore, 662056) and 2.5 μg/μL ubiquitin (Millipore, 662057). Samples were incubated for 2 h at 30 °C and analyzed by immunoblotting.

### Xenograft mouse model

H1975 (5 × 10^5^), PC-9 (2 × 10^6^) or PC-9/AZDR (2 × 10^6^) cells were subcutaneously injected into right flanks of 6-week-old female BALB/C nude mice (*n* = 5 or *n* = 6 per group). Mice were administrated with inhibitors at indicated including erlotinib (100 mg/kg in PBS containing 0.5% glycerol by gastric gavage once daily), osimertinib (5 mg/kg in PBS containing 5% DMSO, 40% PEG 400, and 5% Tween-80 by gastric gavage once daily), ganetespib (50 mg/kg in H_2_O containing 5% DMSO and 45% PEG 400, i.p. once weekly) or nebivolol (10 mg/kg in H_2_O containing 10% DMSO, 40% PEG 400, and 5% Tween-80, i.p. 6 times weekly). Tumor sizes were measured using a caliber twice a week. When applicable, bioluminescence images were captured using IVIS Spectrum (PerkinElmer, Waltham, USA). Tumor weights and volumes (calculated using formula: (length x width^2^)/2) were presented as means ± SEM. Mice were housed in groups of 4–6 mice in an individually ventilated cage (IVC) in a 12:12 light−dark cycle (08:30–20:30 light; 20:30–8:30 dark). The ambient temperature was 22 ± 2 °C with 50–60% relative humidity. All animal care and animal experiments were performed in accordance with the institutional ethical guidelines and were approved by the institutional review board of Sichuan University.

### EGFR^L858R/T790M^-driven lung cancer mouse model

Rosa26-Loxp-Stop-Loxp (LSL)-EGFR^L858R/T790M^ autonomous lung tumor mice were generated by Beijing Biocytogen Co., Ltd. Six-week-old mice were induced for the expression of EGFR^L858R/T790M^ by adenoviruses encoding Cre (Ad-Cre, 2.5 × 10^7^ PFU) via intranasal delivery^55^. At one week and 12 weeks post Cre induction, lentivirus expressing HA-FBXL2 (3 × 10^6^ PFU) or a vector were administrated by intranasal delivery. At 14 weeks after Cre induction, mice were subjected to Micro-CT imaging (Quantum GX II Micro-CT Imaging System, PerkinElmer, Waltham, USA) prior to sacrificed. Images were analyzed by MIM software (MIM Software Inc.).

For nebivolol treatment assay, at 45 days post Cre induction, mice were administrated with nebivolol (10 mg/kg) or vehicle five times weekly for 6 weeks. Five days prior to euthanasia, mice were injected into the tail vein with IRDye 800CM EGF Optical Probe (P/N 926-08446, LI-COR Biosciences, Lincoln, Nebraska, USA) to assess EGFR overexpression on lung tumors by Pearl Trilogy Imagers (LI-COR Biosciences). Lungs were dissected, fixed and paraffin-embedded, and subjected to H&E and IHC staining. All animal care and animal experiments were performed in accordance with the institutional ethical guidelines and were approved by the institutional review board of Sichuan University.

### Immunohistochemistry (IHC) staining

Human tumor tissue array slides (HLugA120PG01 and HLug-Squ150CS-01) were purchased (OUTDO, Shanghai, China). Paraffin-embedded tumors or lung samples were sliced into 4 µm thickness. The slides were subjected to IHC using specific antibodies as indicated. Antibodies were purchased either from Cell Signaling Technology, Abcam or HUABIO (Hangzhou, China), and specific dilutions were indicated: EGFR (CST-4267, 1:100), p-EGFR (CST-3777, 1:200), FBXL2 (ab-153842, 1:100), HA (CST-3724, 1:1000), Ki67 (Ab-15580, 1:200), cleaved caspase-3 (CST-9661, 1:100) and Cyclin D3 (HuaBio, ET1612-4, 1:50). Slides were scanned through NanoZoomer (Hamamatsu, Japan) and the images were captured using the Hamamatsu NDP.view2 viewing software, which was quantified by integrated optical density (IOD) via Image-Pro Plus 6.0 (MD, USA). Average optical density (AOD) was calculated using the formula: AOD = IOD/Area as described^56^.

### Computational virtual screening of putative inhibitors for EGFR

#### Protein structure and compound library preparation

To perfom virtual screening for small molecule inhibitors of FBXO3−FBXL2 interaction, we obtained the target protein structure of the FBXO3-ApaG domain (PDB ID: 5HDW^33^) from Protein Data Bank. The crystal structure was processed by deleting hetero atoms, adding charges, and energy minimization. To create conformational diversity upon protein-ligand binding, a short time (1 ns) molecular simulation was performed on the structure using GROMACS^57^. These simulated conformations were clustered into five modes for the following computation.

2,373 FDA-approved drugs were selected from DrugBank database^34^. The 3D structures of those compounds were predicted by Open Babel^58^ and their hydrogen atoms and partial charges were prepared using MGL Tools^59^.

#### Structure-based virtual screening

The structure-based virtual screening was performed using a hierarchic pipe-line as described^60^. On the first stage, each of the 2,373 small molecule drugs was docked into five individual modes of the ApaG pocket using the rigid docking program DOCK6^61^. The docking region was centered at ILE333 of ApaG pocket with the size of 15 Å in *x*, *y* and *z-*axis as indicated from the docking conformation of BC-1215^26^. The top 30% scored small molecules were selected based on the best DOCK6 scores of each conformational mode. On the secondary stage, the selected small molecules were re-docked using a flexible docking program, AutoDock Vina^62^. Two programs, Vina Score and Cyscore^63^, were used to score protein-ligand binding affinity. The top-scoring 200 compounds were listed in Supplementary Table 2. The top 10 scoring compounds of either program were selected for further inspection. Each compound was analyzed for the possible formation of salt-bridge with GLU341 in the ApaG pocket^64^, which was used as a major feature of BC-1215 binding to ApaG domain^26^. Upon completion of the analyses, three compounds were selected for experimental validations (Supplementary Fig. 8C).

### Biolayer interferometry (BLI)

The binding kinetics and affinity of molecular interactions were detected by Biolayer Interferometry (BLI) using an Octet Red instrument (ForteBio, Inc., Menlo Park, USA) according to the manufacturer’s instructions. Briefly, the purified recombinant EGFR kinase portion was biotinylated and immobilized on streptavidin-coated biosensors and exposed to different concentrations of FBXL2/SKP-1. The binding affinity (*K*_D_) values were calculated using Octet Pro software analysis.

### Bioinformatics analysis

UCSC Xena browser (http://xena.ucsc.edu) was used to analyze the mRNA levels of FBXL2 or Grp94 in LUAD (TCGA Provisional, 524 samples) and LUSC (TCGA Provisional, 501 samples) with paired normal tissues from TCGA datasets.

### Statistical analysis

To perform statistical analysis, at least three independent experiments were performed. Statistical analyses were performed using GraphPad Prism 8 (GraphPad Software). For comparison between two groups, *p* values were determined by two-tailed Student’s *t*-tests, *p < 0.05; **p < 0.01; ***p < 0.001; ns not significant. The correlation coefficients were determined using Pearson’s rank correlation test.

### Reporting summary

Further information on research design is available in the Nature Research Reporting Summary linked to this article.

## Supplementary information

Supplementary Information

Reporting Summary

## Data Availability

The structures of FBXO3-ApaG domain (PDB ID: 5HDW), EGFR kinase domain (PDB 6V66), or FBXL2 (PDB ID: 6O60) were obtained from Protein Data Bank (https://www.rcsb.org/). The FBXL2 expression in TCGA lung cancer datasets was analyzed on the following website: https://portal.gdc.cancer.gov. The KM plotter lung cancer dataset was obtained from http://kmplot.com/analysis. All data generated or analyzed during this study are included in this article and its Supplementary Information files. The uncropped gel or blot figures and original data underlying Figs. 1–7 and Supplementary Figs. 1–11 are provided as a Source Data file. Source data are provided with this paper. All the other data are available within the article and its Supplementary Information. Source data are provided with this paper.

## Source data

Source Data
