# Supplementary material for: FBXL2 counteracts Grp94 to destabilize EGFR and inhibit EGFR-driven NSCLC growth
Source: Nat Commun. 2021 Oct 11;12:5919. doi: 10.1038/s41467-021-26222-x (PMC8505509; doi:10.1038/s41467-021-26222-x)
Supplement: Supplementary file 1 — Supplementary Information [file 41467_2021_26222_MOESM1_ESM.pdf]

Supplementary Information

FBXL2 counteracts Grp94 to destabilize EGFR and inhibit EGFR-driven NSCLC growth

Mengmeng Niu, Jing Xu, *et al.*

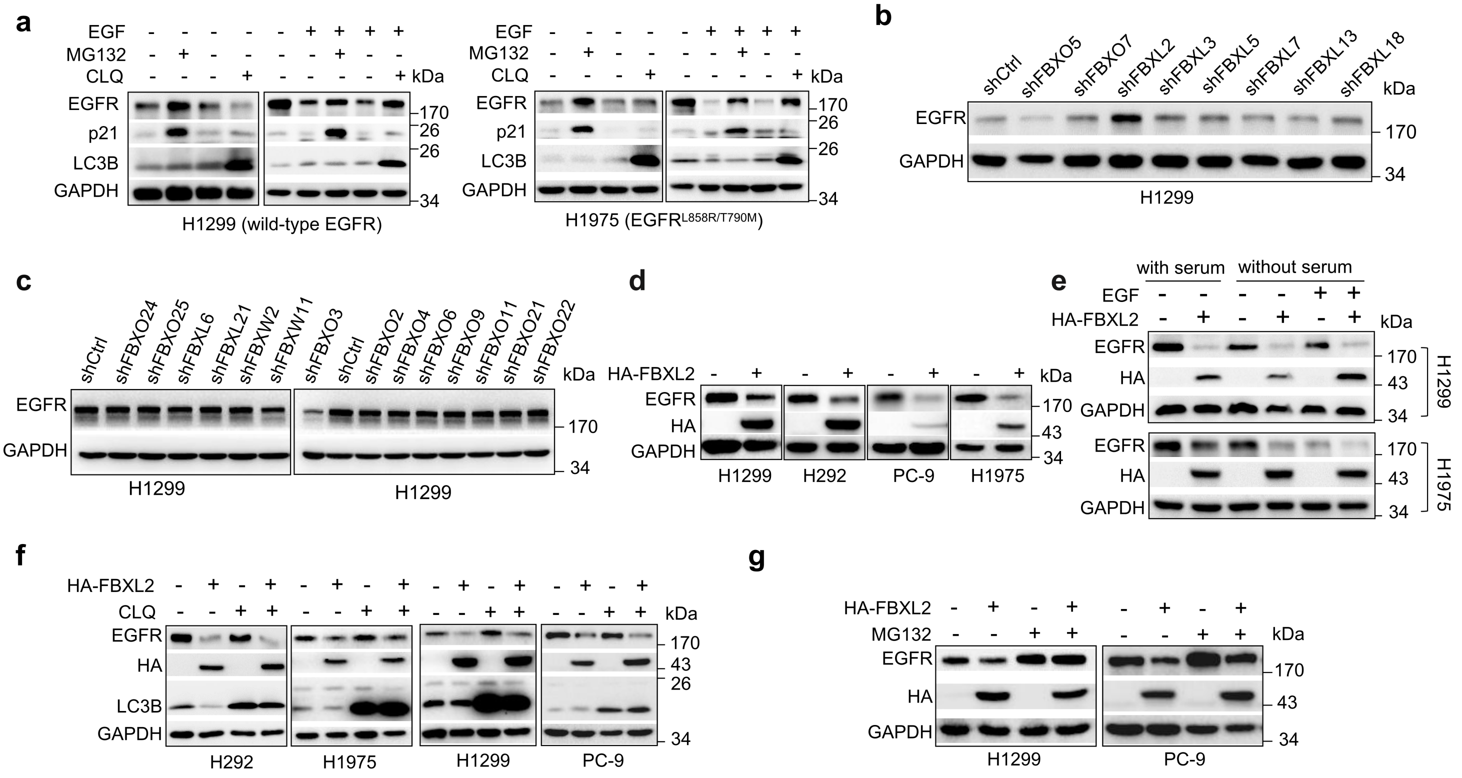

Supplementary Fig. 1. FBXL2 targets EGFR for proteasomal degradation in an EGF-independent manner. (a) H1299 or H1975 cells were grown in serum-free medium for 12 h and treated with 20 μM MG132 for 6 h or 45 μM chloroquine (CLQ) for 24 h. Cells were then treated with or without 100 ng/mL EGF for 10 min prior to Western blot analyses. (b-c) H1299 cells were infected with lentivirus shRNAs mixtures against E3 ligases of F-box family. Cells were then subjected to Western blotting. (d) H1299, H292, PC-9 or H1975 cells stably expressing HA-FBXL2 were subjected to Western blot analyses. (e) FBXL2 inhibits EGFR protein expression independent of EGF stimulation. H1299 or H1975 cells stably expressing HA-FBXL2 were grown with or without serum (10%) for 12 h, and serum-starved cells were then treated with or without 100 ng/mL EGF for 10 min before harvesting for Western blot analyses. (f-g) H1299, H292, PC-9 or H1975 cells stably expressing HA-FBXL2 were treated with MG132 or chloroquine (CLQ) prior to Western blot analyses. The experiment was repeated three times independently with similar results (a-g).

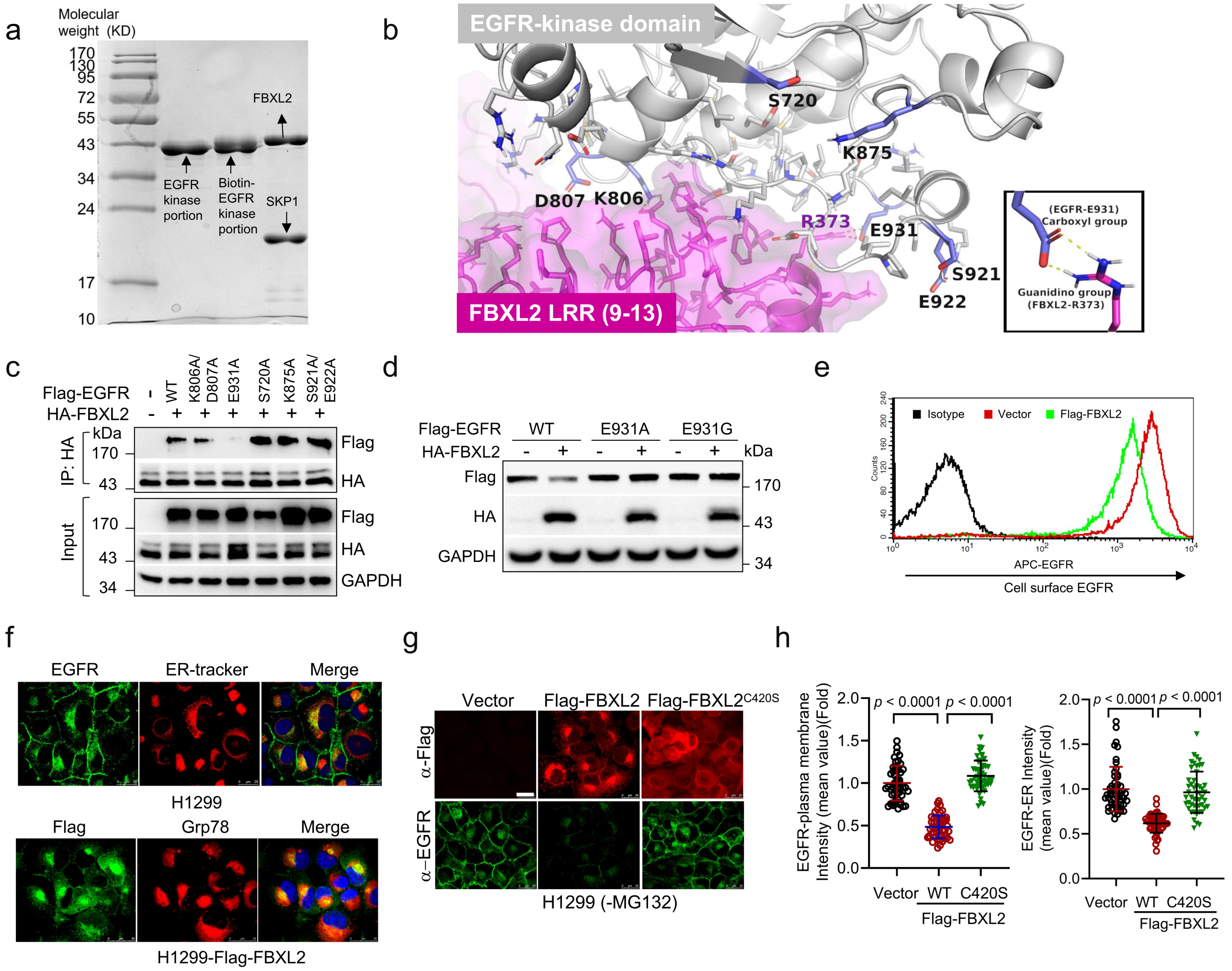

Supplementary Fig. 2. FBXL2 inhibits EGFR protein expression on both plasma membrane and ER. (a) Recombinant FBXL2/SKP1 proteins or recombinant EGFR kinase portion (aa 695-1022; EGFR-K) were purified from SF9 cells. Purified recombinant EGFR kinase portion were biotinylated. (b) The protein-protein interaction prediction tools-ZDOCK 3.0.2 were used to predict the interaction between SKP1-FBXL2 (PDB ID: 6O60) complex and EGFR kinase domain (PDB ID: 6V66). Purple represents 9-13 leucine-rich repeat (LRR) of FBXL2, and Gray represents EGFR kinase domain. (c) HEK293T cells were co-transfected with HA-FBXL2 and indicated Flag-EGFR expressing plasmids. Cells were grown overnight and treated with MG132 for 4 h, followed by IP-Western analyses. (d) HEK293T cells were co-transfected with HA-FBXL2 and indicated Flag-EGFR expressing plasmids for 36 h, and cells were then subjected to Western blot analyses. (e) H292 cells stably expressing Flag-FBXL2 or a vector control were subjected to APC-EGFR staining and FACS analyses. (f) Both EGFR and FBXL2 are localized on the plasma membrane and ER. Upper panel: H1299 cells were co-immunostained for EGFR (Green) and ER tracker (Red), and counterstained with DAPI (Blue). Lower panel: H1299 stably expressing Flag-FBXL2 were co-immunostained for Flag (Green) and Grp78 (Red), and counterstained with DAPI (Blue). (g-h) H1299 cells stably expressing Flag-FBXL2, Flag-FBXL2^{C420S} or a vector control

were subjected to immunostaining for Flag (Red) and endogenous EGFR (Green). The EGFR on plasma membrane and ER were quantified by LAS_X software (n=50 biologically independent cells/group). Data were presented as means ± SD. Scale bar = 25 µm. The experiment was repeated three times independently with similar results (a, c-d and f). Statistical significance was determined by two-tailed Student's *t*-test.

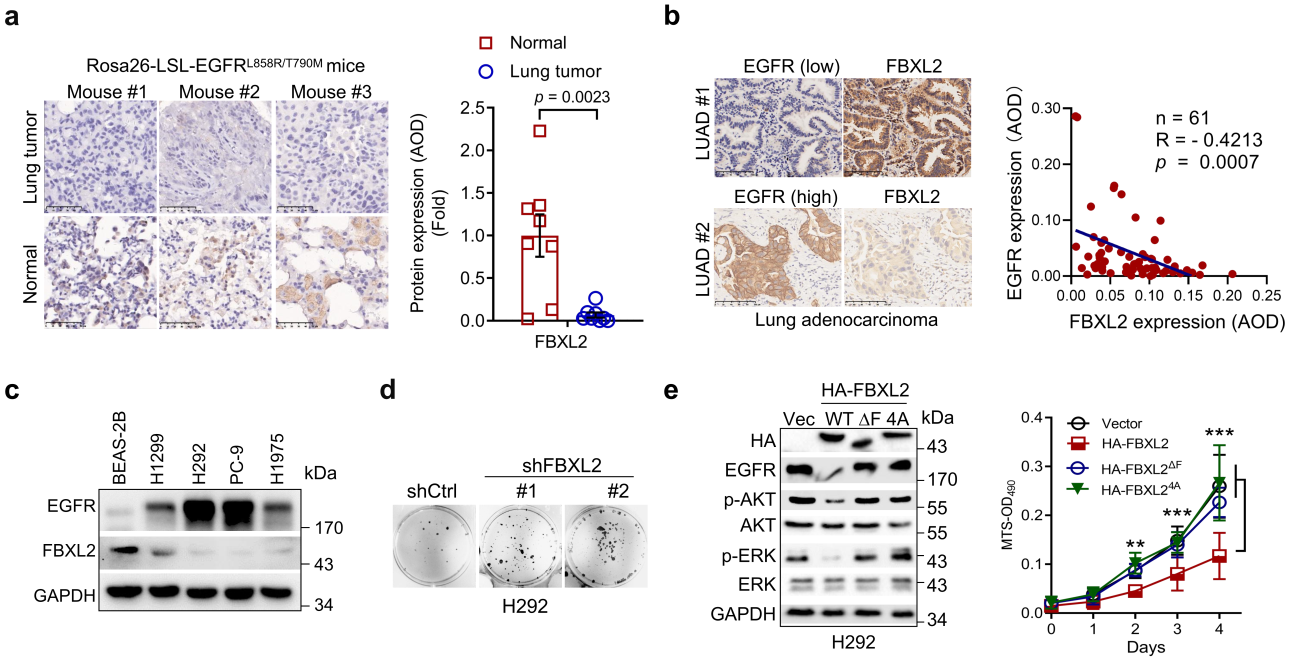

Supplementary Fig. 3. Expression of FBXL2 is reversely correlated with EGFR, and ectopic expression of FBXL2 inhibits cell proliferation. (a) Tumors derived from Rosa26-LSL-EGFR$^{L858R/T790M}$ mice (n=8/group) were subjected to IHC analyses for FBXL2 expression. Data were quantified by AOD and presented as means ± SEM. (b) Expression of FBXL2 is reversely correlated with EGFR in human lung adenocarcinoma (LUAD). Consecutive tissue microarray slides derived from human LUAD were subjected to IHC assay for EGFR and FBXL2 expression and analyzed for Pearson correlation. Staining was quantified by average optical density (AOD). (c) A Reverse correlation of FBXL2 and EGFR in NSCLC cells. Human bronchial epithelial cells BEAS-2B and NSCLC cells H1299, H292, PC-9 or H1975 were subjected to Western blot analyses. Data are representative immunoblots of three independent assays. (d) H292 stable cells as indicated were subjected to Colony formation assays. (e) H292 stable cells as indicated were subjected to immunoblotting or MTS analyses. Three independent experiments were performed. Data were presented as means ± SD. $**p < 0.01$, $***p < 0.001$. Statistical significance was determined by two-tailed Student's *t*-test.

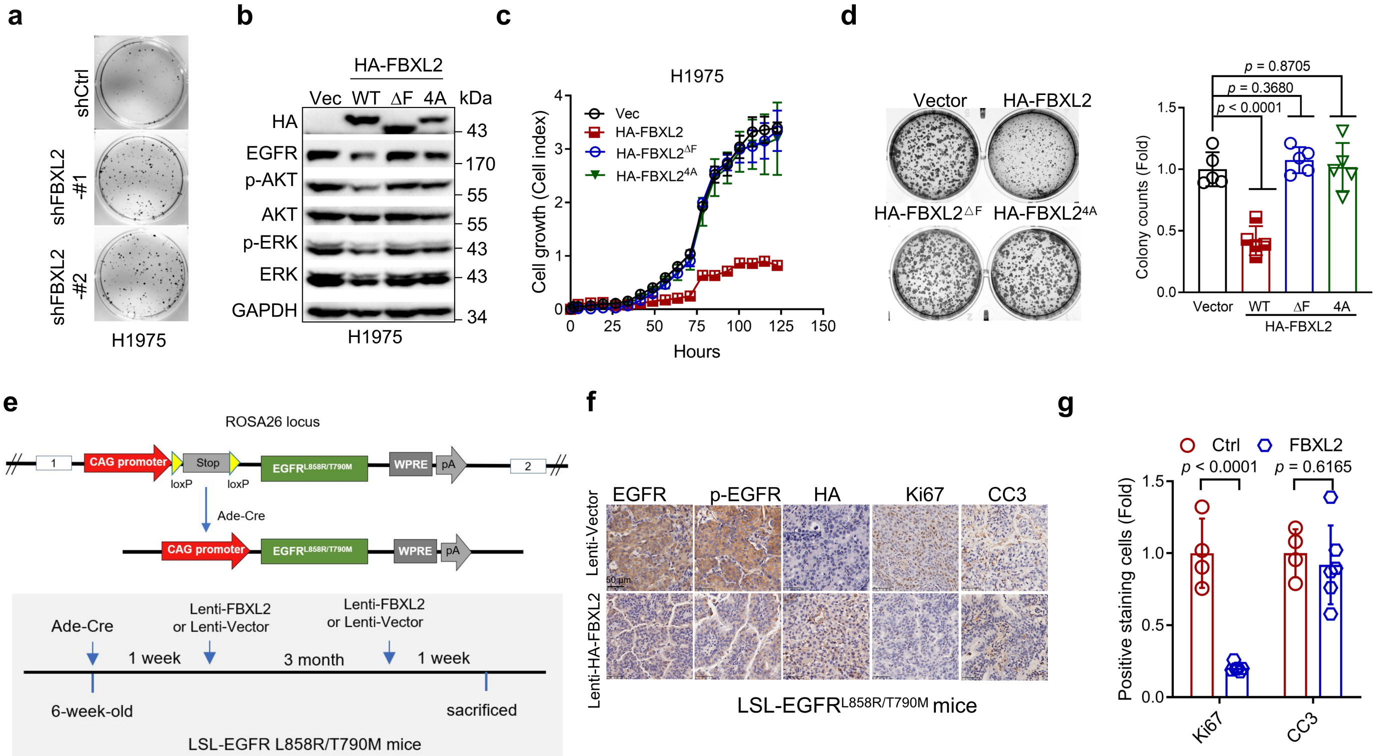

Supplementary Fig. 4. FBXL2 inhibits TKI-resistant NSCLC growth. (a) H1975 stable cells as indicated were subjected to Colony formation assays. (b-d) H1975 cells stably expressing HA-FBXL2 (WT), HA-FBXL2$^{\Delta F}$ or HA-FBXL2^{4A} were subjected to (b) Western blot analyses, (c) Real-Time Cell Analysis (RTCA) (n=2 independent experiments) or (d) Colony formation assays (n=3 independent experiments). Data were presented as means ± SD. (e) A schematic representation of the construct used to generate Rosa26-LSL-EGFR$^{L858R/T790M}$ transgenic mice (C57BL/6) and the experimental procedure to study the effects of HA-FBXL2 on TKI-resistant lung tumor growth. Ade-Cre (2.5x10^{7} PFU); Lenti-HA-FBXL2 (3x10^{6} PFU). (f-g) Lungs shown in Fig. 3o (n=4 or 6/group) were fixed, embedded in paraffin, sectioned and subjected to IHC for EGFR, p-EGFR, HA, Ki67 and cleaved caspase-3 (CC3). Data were quantified by AOD and presented as means ± SEM. Statistical significance was determined by two-tailed Student's *t*-test.

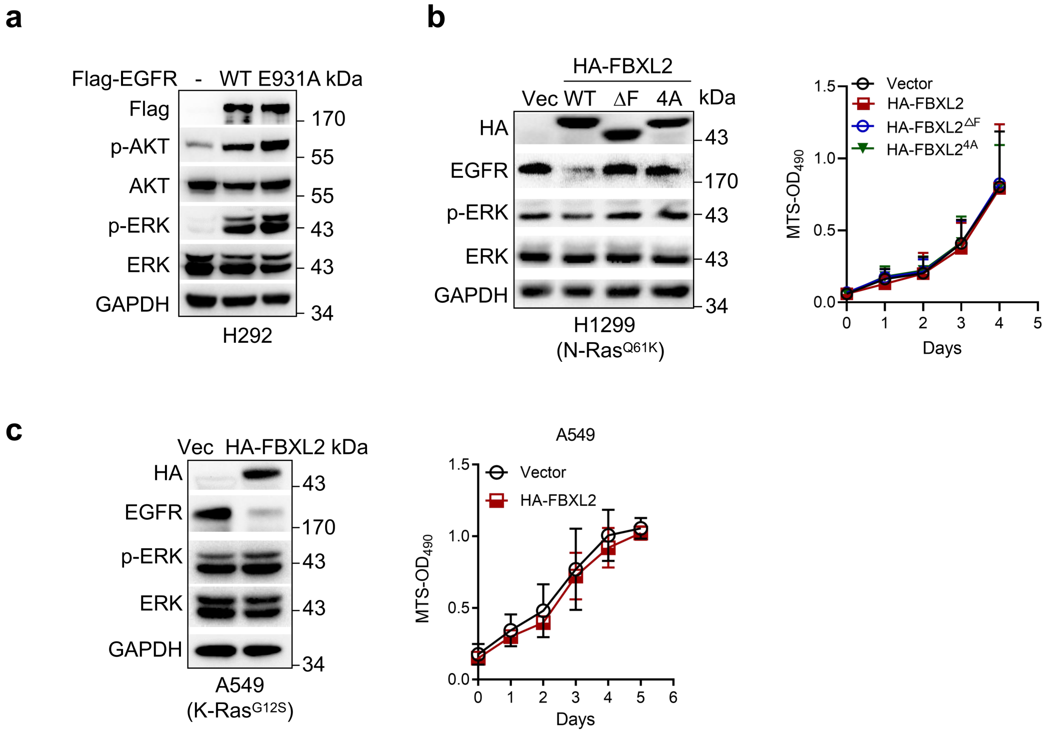

Supplementary Fig. 5. FBXL2 is unable to inhibit proliferation of NSCLC cells harboring activated Ras. (a) $EGFR^{E931A}$ retains EGFR kinase activity. H292 cells stably expressing either Flag-EGFR WT or Flag-$EGFR^{E931A}$ were subjected to Western blot analyses. Data are representative immunoblots of three independent assays. (b-c) A549 (harboring K-Ras^{G12S}) or H1299 (harboring N-Ras^{Q61K}) stable cells as indicated were subjected to immunoblotting and MTS analyses. Three independent experiments in triplicates were performed. Data were presented as means ± SD.

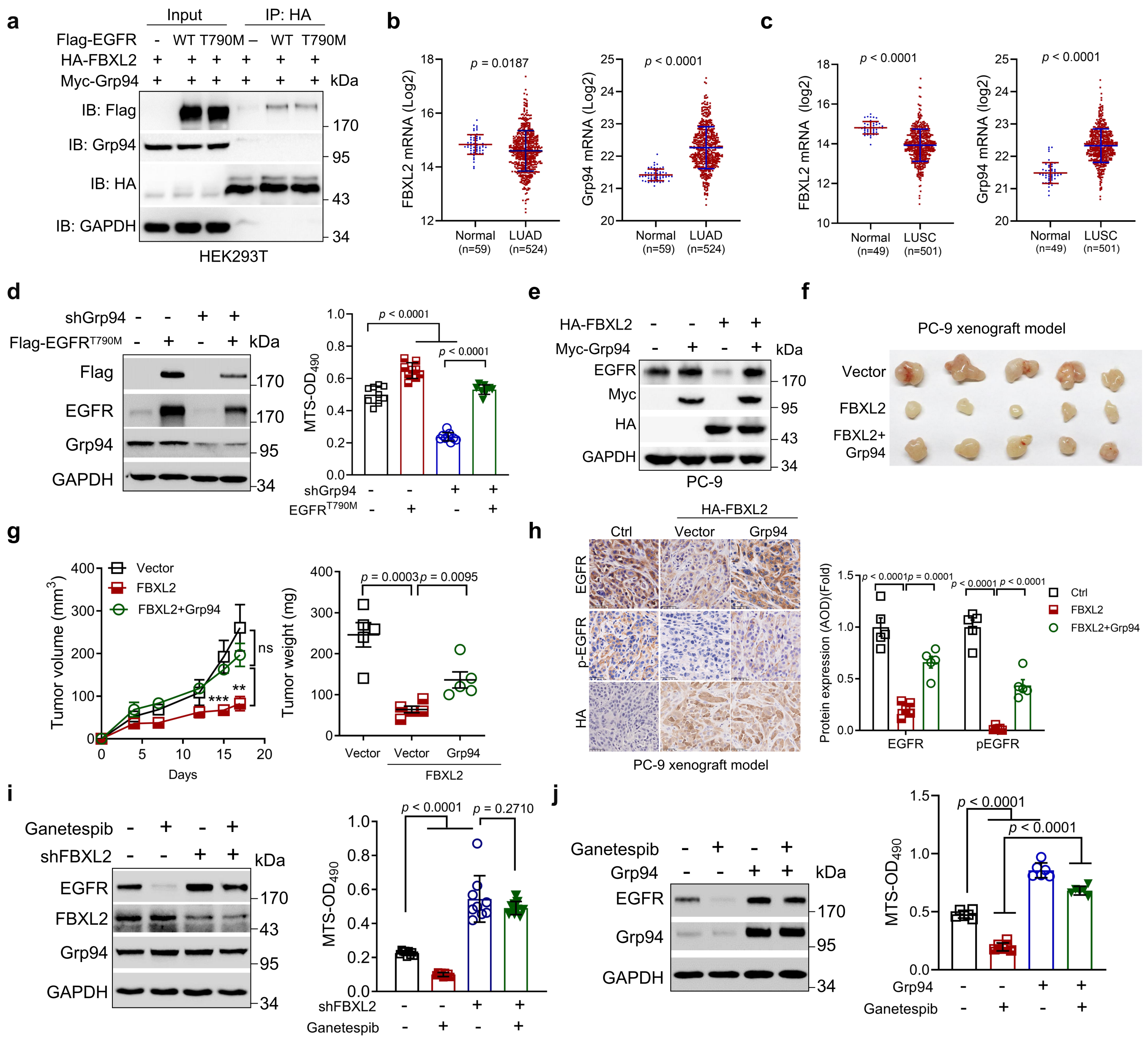

Supplementary Fig. 6. Inhibition of Grp94 suppresses cell proliferation in a FBXL2- or EGFR-dependent manner, and ectopic expression of Grp94 inhibits FBXL2-mediated suppression of NSCLC growth. (a) Grp94 is unable to interaction with FBXL2. HEK293T cells were co-transfected with HA-FBXL2, Myc-Grp94 and indicated Flag-EGFR expressing plasmids for 36 h. Cells were then treated with MG132 for 4 h, followed by IP-Western analyses. Data are representative immunoblots of three independent assays. (b-c) The mRNA levels of FBXL2 and Grp94 in lung adenocarcinoma (LUAD) (n=524) or in human lung squamous cell carcinoma (LUSC) (n=501) with paired normal tissues (n=59 or 49, respectively) were analyzed using the TCGA database. Data were presented as means ± SD. (d) H1975 cells stably silencing of Grp94 were infected with a recombinant lentivirus expressing Flag-EGFR^{T790M} or a vector control, and

cells were then subjected to Western blot analyses and MTS assays. Three independent experiments in triplicates were performed. Data were presented as means ± SD. (e-h) PC-9 cells stably expressing either HA-FBXL2 or Myc-Grp94, or both were subjected to (e) Western blot analyses or (f-h) xenograft tumor growth assays (n=5/group). Photos of tumors, growth curve and tumor weight were presented. Tumors were fixed, embedded in paraffin, sectioned and IHC for EGFR, p-EGFR or HA. Data were quantified by AOD. Data were presented as means ± SEM. $^{**}p = 0.0073$, $^{***}p = 0.0002$. (i-j) H1975 cells stably expressing Grp94 or shRNA against FBXL2 were treated with or without ganetespib (8 nM) for 48 h prior to Western blot analyses and MTS assays. Three independent experiments were performed. Data were presented as means ± SD. Statistical significance was determined by two-tailed Student's t-test.

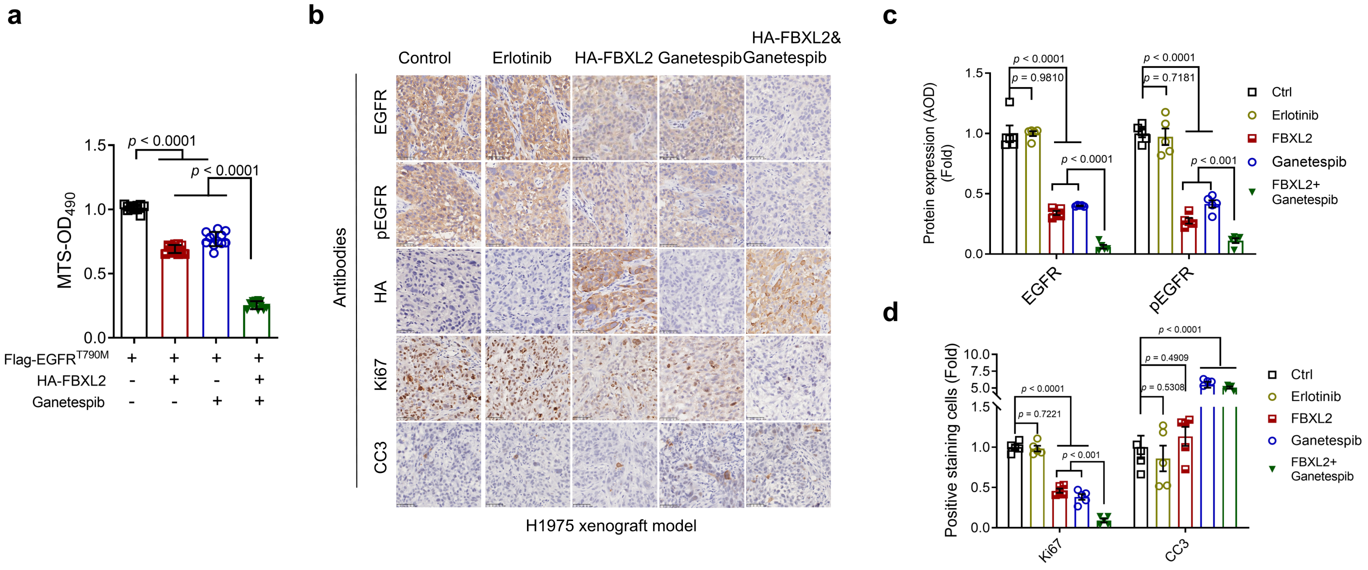

Supplementary Fig. 7. Ganetespib augments FBXL2-induced inhibition of cell proliferation. (a) PC-9 cells stably expressing Flag-EGFR^{T790M} and either HA-FBXL2 or vector were treated with or without ganetespib (8 nM) for 48 h prior to MTS assays. Three independent experiments were performed. Data were presented as means ± SD. (b-d) Tumors shown in Fig. 5R (n=5/group) were fixed, embedded in paraffin, sectioned and IHC for EGFR, p-EGFR, HA, Ki67 and cleaved caspase-3 (CC3). Data were quantified by AOD and presented as means ± SEM. Statistical significance was determined by two-tailed Student's t-test.

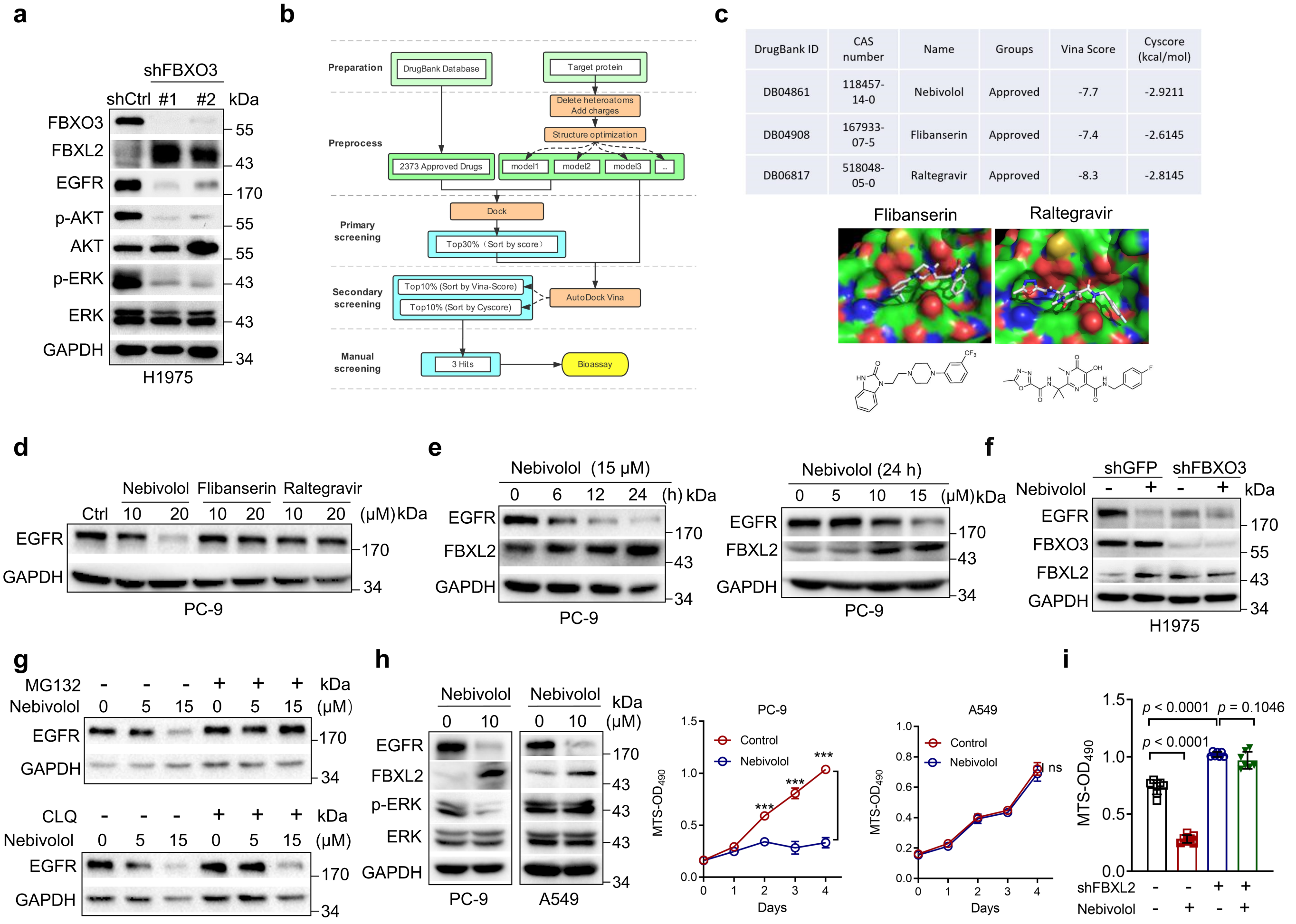

Supplementary Fig. 8. Nebivolol upregulates FBXL2 expression to inhibit cell proliferation in an EGFR- and FBXO3-dependent manner. (a) H1975 cells stably expressing either shFBXO3-1, shFBXO3-2 or a vector control were subjected to Western blot analyses. (b) A flowchart of using FBXO3-ApaG pocket of FBXL2 binding for virtual screening of small inhibitory molecules from DrugBank database. (c) The computer predicted scores (Vina score and Cyscore), the docking diagrams and the chemical structures of three top hits from the screens were shown. (d) PC-9 cells were treated with an indicated dose of nebivolol, flibanserin or raltegravir for 24 h. Cells were then subjected to Western blot analyses. (e) PC-9 cells were treated with an indicated dose of nebivolol for 24 h or treated with 15 μM nebivolol for an indicated time interval prior to Western blot analyses. (f) H1975 stable cells were treated with 15 μM nebivolol for 24 h prior to Western blot analyses. (g) H1975 cells were pre-treated with DMSO, 5 μM or 15 μM nebivolol for 24 h before treated with MG132 for 6 h or chloroquine for 12 h. Cells were then subjected to Western blot analyses. (h) PC-9 or A549 cells were treated with or without 10 μM nebivolol and were then subjected to Western blot analyses (left panel) or MTS assays (right panel). Three independent experiments in triplicates were performed. Data were presented as means ± SD. ***$p < 0.001$. (i) PC-9 stable cells were treated with 8 μM nebivolol for 72 h prior to MTS assays.

Three independent experiments were performed. Data were presented as means ± SD. The experiment was repeated three times independently with similar results (a and d-g). Statistical significance was determined by two-tailed Student's *t*-test.

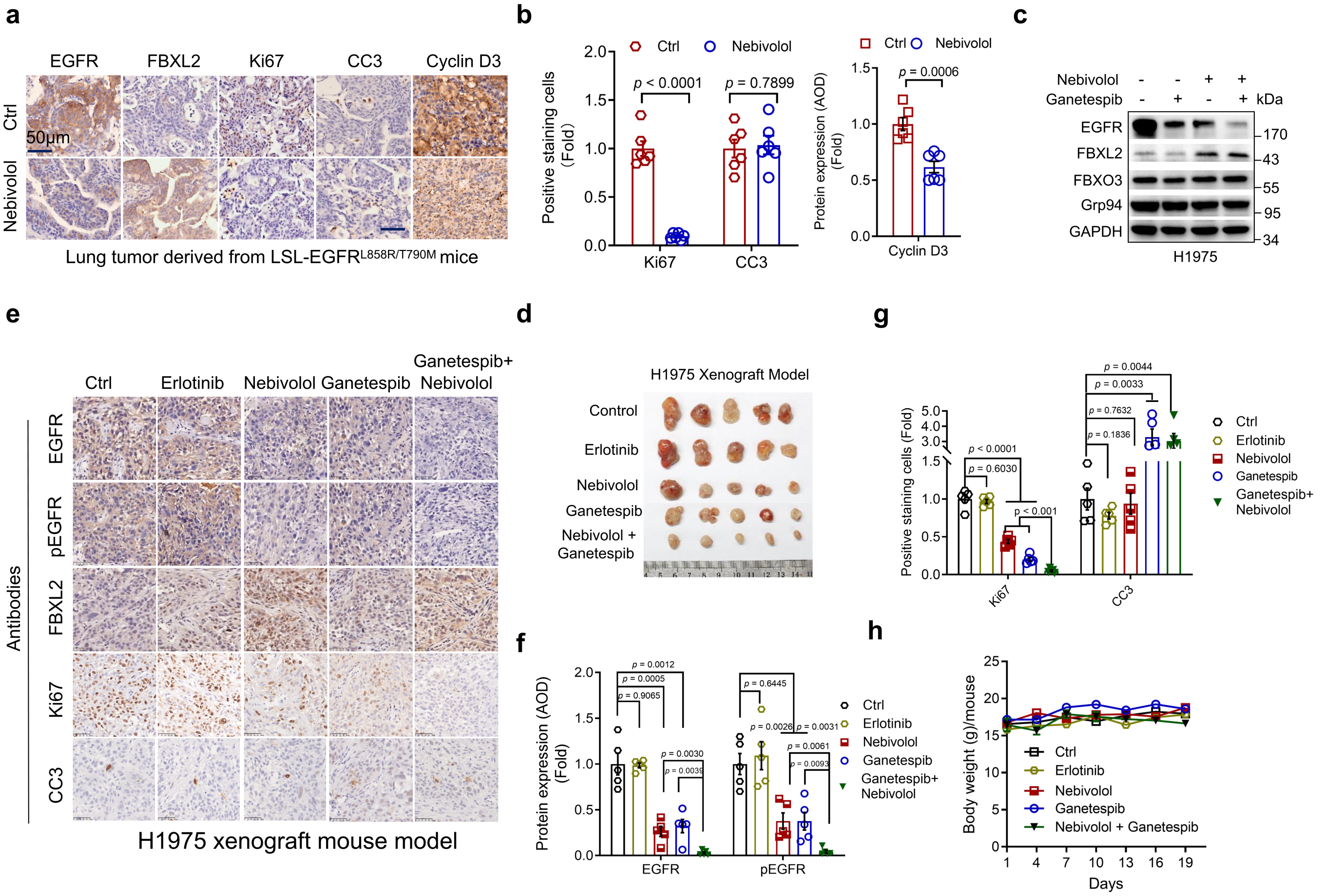

Supplementary Fig. 9. Combination of nebivolol and ganetespib inhibits growth of erlotinib-resistant NSCLC. (a-b) Lungs shown in Fig. 6K (n=6/group) were fixed, embedded in paraffin, sectioned, and IHC was performed for expression of EGFR, FBXL2, Ki67, cleaved caspase-3 (CC3) and cyclin D3. Data were quantified by AOD and presented as means ± SEM. ****p* < 0.001. (c) H1975 cells were treated with nebivolol (10 μM) or ganetespib (8 nM) alone or in combination for 36 h followed by western blot analyses (n=3 independent experiments). (d-h) H1975 cells ($5x10^5$) were subjected to xenograft tumor growth assays (n=5/group). Tumors were fixed, embedded in paraffin, sectioned, and IHC was performed for expression of EGFR, p-EGFR (Try1068), FBXL2, Ki67 and cleaved caspase-3 (CC3). Body weight of mice shown were measured. Data were quantified by AOD and presented as means ± SEM. Statistical significance was determined by two-tailed Student's *t*-test.

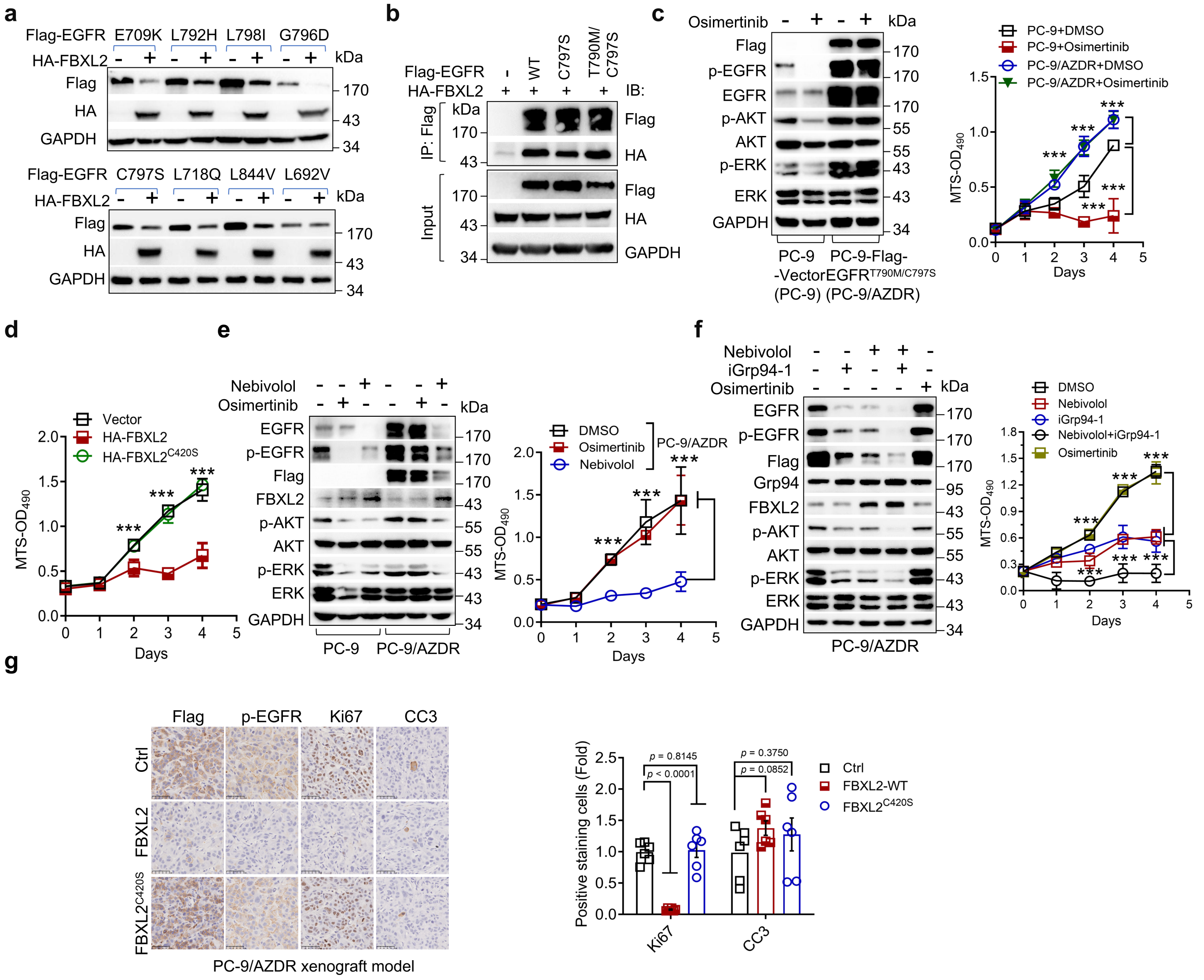

Supplementary Fig. 10. Activation of FBXL2 inhibits osimertinib-resistant cell proliferation, which is augmented by inhibition of Grp94. (a) HEK293T cells were co-transfected with HA-FBXL2 and either wild-type Flag-EGFR or an indicated Flag-EGFR mutant expressing plasmids for 36 h, followed by Western blot analyses (n=3 independent experiments). (b) HEK293T cells were co-transfected with FBXL2 and an indicated EGFR expressing plasmids. Cells were grown overnight and treated with 20 μM MG132 for 4 h, followed by IP-Western analyses (n=3 independent experiments). (c) PC-9 cells stably expressing Flag-EGFR$^{T790M/C797S}$ (PC-9-Flag-EGFR$^{T790M/C797S}$) were treated with 6 nM osimertinib for 36 h, and then subjected to Western blot analyses or MTS assays. Three independent experiments in triplicates were performed. Data were presented as means ± SD. ***$p < 0.001$. (d) PC-9-Flag-EGFR$^{T790M/C797S}$ cells stably expressing HA-FBXL2 or HA-FBXL2^{C420S} were subjected to MTS assays. Three independent experiments were performed. Data were presented as means ± SD. ***$p < 0.001$. (e) PC-9 cells stably expressing Flag-EGFR$^{T790M/C797S}$

or a vector control were treated with osimertinib (6 nM) or nebivolol (10 μM) for 36 h or an indicated time interval, followed by Western blot analyses or MTS assays. Three independent experiments in triplicates were performed. Data were presented as means ± SD. ***$p < 0.001$. (f) PC-9 cells stably expressing Flag-EGFR$^{T790M/C797S}$ were treated with nebivolol (10 μM) or Grp94 inhibitor-1 (iGrp94-1, 15 μM) alone or in combination for 36 h or an indicated time interval, followed by Western blot analyses or MTS assays. Three independent experiments in triplicates were performed. Data were presented as means ± SD. ***$p < 0.001$. (g) Tumors shown in Fig. 7E (n=6/group) were fixed, embedded in paraffin, sectioned and subjected to IHC for Flag (EGFR), p-EGFR, Ki67 and cleaved caspase-3 (CC3). Data were quantified by AOD and presented as means ± SEM. Statistical significance was determined by two-tailed Student's *t*-test.

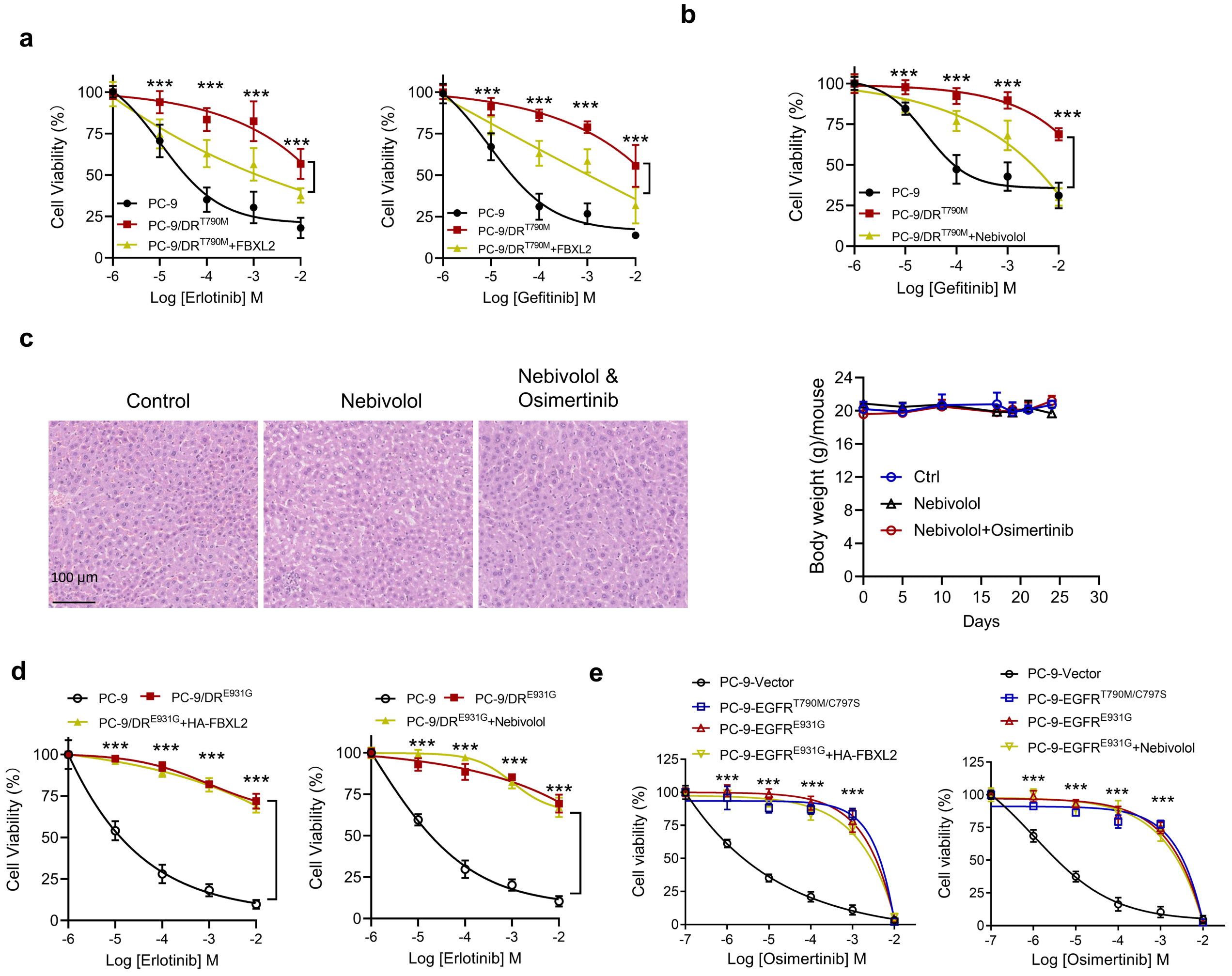

Supplementary Fig. 11. Activation of FBXL2 overcomes PC-9/DR^{T790M}-induced erlotinib or gefitinib resistance, but it has little effects on PC-9/DR^{E931G}-induced resistance to erlotinib, gefitinib or osimertinib. (a) PC-9 cells expressing TKI-resistant EGFR^{T790M} (PC-9/DR^{T790M})

were infected with lentivirus encoding HA-FBXL2 or a vector control. Cells were treated with an indicated dose of erlotinib (Left panel) or gefitinib (Right panel) for 72 h prior to MTS assays. Data derived from three independent experiments in triplicates were presented as means ± SD. ***$p < 0.001$. (b) PC-9 or PC-9/DR^{T790M} cells were treated with an indicated dose of gefitinib in the absence or presence of 5 μM nebivolol for 48 h followed by MTS assays. Data from three independent experiments were presented as means ± SD. ***$p < 0.001$. (c) The liver of mice shown in Fig. 7J (n=5/group) were subjected to H&E staining and body weight of mice were measured. Data presented as means ± SD. (d) Left panel: PC-9 cells expressing erlotinib-resistant EGFR^{E931G} (PC-9/DR^{E931G}) were infected with lentivirus encoding HA-FBXL2 or a vector control. Cells were treated with an indicated dose of erlotinib for 72 h prior to MTS analyses. Right panel: PC-9 cells expressing erlotinib-resistant EGFR^{E931G} (PC-9/DR^{E931G}) were treated with an indicated dose of erlotinib in the absence or presence of 5 μM nebivolol for 72 h followed by MTS assays. Data derived from three independent experiments in triplicates were presented as means ± SD. ***$p < 0.001$. (e) Left panel: PC-9 cells expressing EGFR$^{T790M/C797S}$ (PC-9-EGFR$^{T790M/C797S}$) or EGFR^{E931G} (PC-9-EGFR^{E931G}) were infected with lentivirus encoding HA-FBXL2 or a vector control. Cells were treated with an indicated dose of osimertinib for 72 h prior to MTS analyses. Right panel: PC-9 cells expressing EGFR$^{T790M/C797S}$ (PC-9-EGFR$^{T790M/C797S}$) or EGFR^{E931G} (PC-9-EGFR^{E931G}) were treated with an indicated dose of osimertinib in the absence or presence of 5 μM nebivolol for 72 h followed by MTS assays. Data derived from three independent experiments in triplicates were presented as means ± SD. ***$p < 0.001$. Statistical significance was determined by two-tailed Student's *t*-test.

Supplementary Table 1. Primers used in this study

Gene name	primer sequence
Human shFBXL2-1	F: CCGGGCACAGATTTCCAAGGCTTTTCTCGAGAAAAGCCTTGGAAATCTGTGCTTTTTG<br>R: AATTCAAAAAGCACAGATTTCCAAGGCTTTTCTCGAGAAAAGCCTTGGAAATCTGTGC
Human shFBXL2-2	F: CCGGGCCTTTCGGGTTGCAGCAATTCTCGAGAATTGCTGCAACCCGAAAGGCTTTTTG<br>R: AATTCAAAAAGCCTTTCGGGTTGCAGCAATTCTCGAGAATTGCTGCAACCCGAAAGGC
Human shFBXL2-3	F: CCGGGCTCGGAATTGCCACGAATCTCGAGATTCGTGGCAATTCCGAGCTTTTTG<br>R: AATTCAAAAAGCTCGGAATTGCCACGAATCTCGAGATTCGTGGCAATTCCGAGC
Human shFBXO3-1	F: CCGGAGGAAGATACATTGACCATTACTCGAGTAATGGTCAATGTATCTTCCTTTTTTG<br>R: AATTCAAAAAAGGAAGATACATTGACCATTACTCGAGTAATGGTCAATGTATCTTCCT
Human shFBXO3-2	F: CCGGCCTGGGTTCTATGTGACACTACTCGAGTAGTGTCACATAGAACCCAGGTTTTTG<br>R: AATTCAAAAATGGGTTCTATGTGACACTACTCGAGTAGTGTCACATAGAACCCAGG
Human shGrp94-1	F: CCGGTCGCCTCAGTTTGAACATTGATTCAAGAGATCAATGTTCAAACTGAGGCGATTTTTG<br>R: AATTCAAAAATCGCCTCAGTTTGAACATTGATCTCTTGAATCAATGTTCAAACTGAGGCGA
Human shGrp94-2	F: CCGGAAGTTGATGTGGATGGTACATTCAAGAGATGTACCATCCACATCAACTTTTTTTG<br>R: AATTCAAAAAAAGTTGATGTGGATGGTACATCTCTTGAATGTACCATCCACATCAACTT
Human shEGFR	F: CCGGCGCAAAGTGTGTAACGGAATA CTCGAG TATTCCGTTACACACTTTGCG TTTTTG<br>R: AATTCAAAAACGCAAAGTGTGTAACGGAATA CTCGAG TATTCCGTTACACACTTTGCG
Human FBXL2	F: GCTCTAGAATGGTTTTCTCAAACAATGATGAAGGC<br>R: TTGCGGCCGCTCAGAGAATGACACA
Human FBXL2-ΔF	F: GATCTTTTTAACTTTCAAACAG'<br>R: TTCATCATTGTTTGAGAAAAC
Human FBXL2-4AW	3A-F: GCAGCCGCAGAACTTCTGTTAAGAATATTTTCCTTC<br>3A-R: CTTTTTGTTAATAAGGCCTTCATCATTGTTTGAGA<br>W-F: GCGAACATCTTAGCCCTGGATGGAAGCAAC<br>W-R: AGCCTTGGAAATCTGTGCACATCGGCACAAAG
Human FBXL2-C420S	F: TCTGTCATTCTCTGAGCGGCCGCCCCGGGT<br>R: GCACCTGCACAGTCGCTGTCCACTTCCT
Human FBXL2 Δ1-4 LRR	F: TATTCTTTGCCAGTTGCTTCCATCCA<br>R: TGTGATCAGATCACGAAGGATGGCA
Human FBXL2 Δ5-8 LRR	F: CCAAGAGAGGTTCAGGTACTCCAGG<br>R: TGCTCCCATTTGACTGACGCAG
Human FBXL2 Δ9-13 LRR	F: TCGGGCAGCCTCCAAAATTTGCAGT<br>R: CCCGTCACCCCACCGACAGCAGT

Human EGFR	F: CGGAATTCGCCACCATGCGACCCTCCGGGACGGCCGGGGCAGCGCTCCTG<br>R: TTGCGGCCGCCTACTTGTCATCGTCATCCTTGTAATCACCTGCTCCAATAAATTC
Human EGFR L718Q	F: CAGGGCTCCGGTGCGTTCGGCAC<br>R: CACTTTGATCTTTTTGAATTCAGTTTCCTT
Human EGFR L844V	F: GTGGTGAAAACACCGCAGCATGTCAA<br>R: TACGTTCCTGGCTGCCAGGTCGCGG
Human EGFR L798I	F: ATCCTGGACTATGTCCGGGAACAC<br>R: GCAGCCGAAGGGCATGAGCTGCGT
Human EGFR G796D	F: GACTGCCTCCTGGACTATGTCCGGG<br>R: GAAGGGCATGAGCTGCGTGATGAG
Human EGFR L692V	F: GTTACACCCAGTGGAGAAGCTCCC<br>R: AGGCTCCACAAGCTCCCTCTCCTGC
Human EGFR E709K	F: AAAACTGAATTCAAAAAGATCAAAGTG<br>R: CTTCAAGATCCTCAAGAGAGCTTG
Human EGFR L792H	F: CACATGCCCTTCGGCTGCCTCCTGGAC<br>R: CTGCGTGATGAGCTGCACGGTGGAGG
Human EGFR C797S	F：TCCCTCCTGGACTATGTCCGGGAA<br>R：GCCGAAGGGCATGAGCTGCGTGAT
Human EGFR-ΔJx	F: ACGC GTCGACGTCGAAGGCGCCACATCGTTCGGA<br>R: ATAAGAATGCGGCCGCTCAGAATTCAGTTTCCTTCA
Human EGFR-ΔK	F: ACGCGTCGACGTAAAAAGATCAAAGTGCTGGGCT<br>R: ATAAGAATGCGGCCGCTCAAATGACAAGGTAGCGC
Human EGFR-ΔC	F: ACGCGTCGACGTCAGGGGGATGAAAGAATGC<br>R: ATAAGAATGCGGCCGCTCATGCTCCAATAAATTCA
Human EGFR-ΔKC	F: ACGCGTCGACGTAAAAAGATCAAAGTGCTGGGCT<br>R: ATAAGAATGCGGCCGCTCATGCTCCAATAAATTCA
Human EGFR-Kinase	F: TAGAGGATCTATTTCCGGTGGCCACCATG AAAAAGATCAAAGTGCTGGGCT<br>R:CGGCCAAAGTGGACCCGGGGCCTACTTGTCATCGTCATCCTTGTAATCACCAATGACAAG GTAGCGC
Human EGFR-C	F: TAGAGGATCTATTTCCGGTGGCCACCATG CAGGGGGATGAAAGAATGC<br>R: CGGCCAAAGTGGACCCGGGGCCTACTTGTCATCGTCATCCTTGTAATCACCTGCTCCAAT AAATTCA
Human EGFR-S720A	F：GCCGGTGCGTTCGGCACGGTGTAT<br>R：GCCCAGCACTTTGATCTTTTTGAATT
Human EGFR	F: GCAGTGCCTATCAAGTGGATGGCATT

K875A	R: GCCTCCTTCTGCATGGTATTCTTTCT
Human EGFR E931A	F: GCACGCCTCCCTCAGCCACCCATAT<br>R: TCCTTTCTCCAGGATGGAGGAGATCTC
Human EGFR S921A/E922A	F: GCCGCGATCTCCTCCATCCTGGAGAAA<br>R: GGCAGGGATTCCGTCATATGGCTTGGA
Human EGFR K806A/D807A	F: GCAGCCAATATTGGCTCCCAGTACCTG<br>R: GTGTTCCCGGACATAGTCCAGGAGG
Human Grp94	F: TCTACTAGAGGATCTATTTCCGGTGGCCACCATGAGGGCCCTGTGGGTGCT<br>R: AAGTGGACCCGGGGCCTACAGATCCTCTTCTGAGATGAGTTTTTGTTCCAATTCATCTTT TTCAGCTGTAG

Supplementary Table 2. The binding scores (Autodock Vina) of the compounds binding to FBXO3-ApaG domain.

DrugBank ID	Score	DrugBank ID	Score	DrugBank ID	Score	DrugBank ID	Score
DB03326	−8.3	DB08126	−7.7	DB01100	−7.4	DB08513	−7.3
DB01988	−8.3	DB00320	−7.7	DB01026	−7.4	DB08865	−7.3
DB02449	−8.2	DB06977	−7.6	DB07817	−7.4	DB07294	−7.3
DB08237	−8.2	DB03383	−7.6	DB01691	−7.4	DB08429	−7.3
DB08091	−8.2	DB07333	−7.6	DB01067	−7.4	DB07264	−7.3
DB06938	−8.2	DB07360	−7.6	DB02741	−7.4	DB07054	−7.3
DB00872	−8.2	DB03802	−7.6	DB08450	−7.4	DB03878	−7.3
DB06896	−8.2	DB01251	−7.6	DB03532	−7.4	DB07075	−7.3
DB06684	−8.2	DB09073	−7.6	DB07382	−7.4	DB07334	−7.3
DB06925	−8.1	DB07549	−7.6	DB07124	−7.4	DB00398	−7.3
DB06962	−8.1	DB03325	−7.6	DB00734	−7.4	DB04432	−7.3
DB08073	−8.1	DB07536	−7.6	DB07031	−7.4	DB07456	−7.3
DB01897	−8.1	DB01003	−7.6	DB07458	−7.4	DB07672	−7.3
DB06963	−8.1	DB04452	−7.6	DB01061	−7.4	DB08749	−7.3
DB03782	−8.0	DB02551	−7.6	DB02573	−7.4	DB07783	−7.2
DB06976	−8.0	DB04714	−7.6	DB04861	−7.4	DB07664	−7.2
DB08003	−8.0	DB07970	−7.6	DB02491	−7.4	DB04722	−7.2
DB07545	−8.0	DB06882	−7.6	DB07256	−7.4	DB07787	−7.2
DB09053	−8.0	DB07508	−7.6	DB00251	−7.4	DB07247	−7.2
DB02354	−8.0	DB08358	−7.6	DB08822	−7.4	DB08064	−7.2
DB07220	−7.9	DB07362	−7.6	DB00222	−7.4	DB01212	−7.2
DB06959	−7.9	DB08221	−7.6	DB07320	−7.4	DB02932	−7.2
DB07397	−7.9	DB04632	−7.6	DB06840	−7.3	DB03696	−7.2
DB07041	−7.9	DB03957	−7.5	DB07583	−7.3	DB07268	−7.2

DB08384	−7.9	DB04591	−7.5	DB07460	−7.3	DB08400	−7.2
DB07651	−7.9	DB07010	−7.5	DB03044	−7.3	DB08487	−7.2
DB04760	−7.9	DB07189	−7.5	DB03337	−7.3	DB03571	−7.2
DB01831	−7.8	DB01267	−7.5	DB06908	−7.3	DB04946	−7.2
DB08730	−7.8	DB04908	−7.5	DB07630	−7.3	DB06995	−7.2
DB07528	−7.8	DB02269	−7.5	DB07966	−7.3	DB04791	−7.2
DB00619	−7.8	DB08568	−7.5	DB08164	−7.3	DB02547	−7.2
DB02705	−7.8	DB01411	−7.5	DB07186	−7.3	DB02181	−7.2
DB08512	−7.8	DB00210	−7.5	DB08366	−7.3	DB08495	−7.2
DB07025	−7.8	DB09195	−7.5	DB08893	−7.3	DB07066	−7.2
DB07406	−7.8	DB07026	−7.5	DB07107	−7.3	DB01940	−7.2
DB06997	−7.8	DB01761	−7.5	DB09272	−7.3	DB04097	−7.1
DB07159	−7.8	DB02258	−7.5	DB00303	−7.3	DB06817	−7.1
DB04759	−7.8	DB04792	−7.5	DB02723	−7.3	DB07649	−7.1
DB00696	−7.7	DB04868	−7.5	DB07156	−7.3	DB01254	−7.1
DB07789	−7.7	DB07605	−7.5	DB09199	−7.3	DB07255	−7.1
DB02830	−7.7	DB02473	−7.5	DB07337	−7.3	DB08930	−7.1
DB04494	−7.7	DB06809	−7.5	DB07422	−7.3	DB07340	−7.1
DB03466	−7.7	DB06888	−7.5	DB07991	−7.3	DB08079	−7.1
DB07204	−7.7	DB06210	−7.5	DB01767	−7.3	DB01459	−7.0
DB07076	−7.7	DB07586	−7.5	DB07423	−7.3	DB06589	−7.0
DB03231	−7.7	DB08815	−7.5	DB07743	−7.3	DB04107	−6.9
DB01184	−7.7	DB07809	−7.5	DB08124	−7.3		
DB08426	−7.7	DB02106	−7.5	DB03907	−7.3		
DB07145	−7.7	DB04764	−7.5	DB07728	−7.3		
DB08044	−7.7	DB08896	−7.4	DB07261	−7.3		
