# Supplementary material for: FBXL2 counteracts Grp94 to destabilize EGFR and inhibit EGFR-driven NSCLC growth
Source: Nat Commun. 2021 Oct 11;12:5919. doi: 10.1038/s41467-021-26222-x (PMC8505509; doi:10.1038/s41467-021-26222-x)
Supplement: Supplementary file 2 — Reporting Summary [file 41467_2021_26222_MOESM2_ESM.pdf]

nature research

Corresponding author(s): Yang Cao and Zhi-Xiong Jim Xiao

Last updated by author(s): Aug 23, 2021

Reporting Summary

Nature Research wishes to improve the reproducibility of the work that we publish. This form provides structure for consistency and transparency in reporting. For further information on Nature Research policies, see our Editorial Policies and the Editorial Policy Checklist.

Statistics

For all statistical analyses, confirm that the following items are present in the figure legend, table legend, main text, or Methods section.

n/a	Confirmed	
☐	☒	The exact sample size (*n*) for each experimental group/condition, given as a discrete number and unit of measurement
☐	☒	A statement on whether measurements were taken from distinct samples or whether the same sample was measured repeatedly
☐	☒	The statistical test(s) used AND whether they are one- or two-sided<br>*Only common tests should be described solely by name; describe more complex techniques in the Methods section.*
☒	☐	A description of all covariates tested
☐	☒	A description of any assumptions or corrections, such as tests of normality and adjustment for multiple comparisons
☐	☒	A full description of the statistical parameters including central tendency (e.g. means) or other basic estimates (e.g. regression coefficient) AND variation (e.g. standard deviation) or associated estimates of uncertainty (e.g. confidence intervals)
☐	☒	For null hypothesis testing, the test statistic (e.g. *F*, *t*, *r*) with confidence intervals, effect sizes, degrees of freedom and *P* value noted<br>*Give P values as exact values whenever suitable.*
☒	☐	For Bayesian analysis, information on the choice of priors and Markov chain Monte Carlo settings
☒	☐	For hierarchical and complex designs, identification of the appropriate level for tests and full reporting of outcomes
☐	☒	Estimates of effect sizes (e.g. Cohen's *d*, Pearson's *r*), indicating how they were calculated

Our web collection on statistics for biologists contains articles on many of the points above.

Software and code

Policy information about availability of computer code

Data collection	NDP.view2 viewing software to IHC image data collection;<br>Image lab 3.0 to western blot data collection;<br>Quantum GX II Micro-CT Imaging System to Micro-CT data collection;<br>Pearl Trilogy Imagers (LI-COR Biosciences) to assess EGFR expression on lung tumors data collection.
Data analysis	Image-Pro Plus 6.0 (MD, USA) to quantify IHC staining by integrated optical density (IOD);<br>GraphPad 8.0 and Excel to do the graph figures and statistics;<br>MIM software (7.0.7, MIM Software Inc., Beijing, China) to analysis the Micro-CT images;<br>Octet Pro software analysis (10.0.1.6) to calculated the binding affinity (KD) value;<br>Vina Score (1.1.2) and Cyscore (2.0) to score protein-ligand binding affinity.<br>LAS_X software (23.0) to analysis fluorescent images.<br>Gromacs software 5.0.7 was used to a short time (1 ns) molecular simulation on the structure

For manuscripts utilizing custom algorithms or software that are central to the research but not yet described in published literature, software must be made available to editors and reviewers. We strongly encourage code deposition in a community repository (e.g. GitHub). See the Nature Research guidelines for submitting code & software for further information.

Data

Policy information about availability of data

All manuscripts must include a data availability statement. This statement should provide the following information, where applicable:

- Accession codes, unique identifiers, or web links for publicly available datasets
- A list of figures that have associated raw data
- A description of any restrictions on data availability

The structures of FBXO3-ApaG domain (PDB ID: 5HDW), EGFR kinase domain (PDB 6V66), or FBXL2 (PDB ID: 6O60) were obtained from Protein Data Bank (https://www.rcsb.org/). The FBXL2 expression in TCGA lung cancer datasets was analyzed on the following website: https://portal.gdc.cancer.gov. The KM plotter lung cancer dataset was obtained from http://kmplot.com/analysis. All data generated or analyzed during this study are included in this article and its Supplementary Information files. The uncropped gel or blot figures and original data underlying Figs. 1–7 and Supplementary Figs. 1–11 are provided as a Source Data file. Source data are provided with this paper. All data supporting the findings of this study are available from the corresponding authors upon reasonable request.

Field-specific reporting

Please select the one below that is the best fit for your research. If you are not sure, read the appropriate sections before making your selection.

☒ Life sciences ☐ Behavioural & social sciences ☐ Ecological, evolutionary & environmental sciences

For a reference copy of the document with all sections, see nature.com/documents/nr-reporting-summary-flat.pdf

Life sciences study design

All studies must disclose on these points even when the disclosure is negative.

Sample size	For in vitro experiments, at least three biologically independent experiments were performed for all experiments unless otherwise stated. No statistical method was used to predetermine sample size. Such sample sizes are typical for the in vitro experiments and sufficient for a statistical analysis. For in vivo experiments, a sample size of n = 4-6 mice per group were used, which is sufficient to generate statistically significant results. No statistical method was used to predetermine sample size. The sample size for animal studies were designed according to previous report (PMID: 24250214) and were chosen based on prior experience with the same experimental design.
Data exclusions	No data were excluded from the analyses
Replication	All attempts for replication were successful by different co-authors of this study. For in vitro experiments, at least three biologically independent experiments were performed for all experiments unless otherwise stated. For in vivo experiments, n=4-6 mice/group mice were used.
Randomization	Yes, mice were randomly divided into different experimental groups and housed under standard conditions.
Blinding	The lab technician who measured the mice were blinded to the treatment groups, and experimenters who performed IHC analyses were blinded to group allocation. We performed other experiments, such as Western blotting and MTS assays, in a non-blinded manner, since the experimental design was complicated and the researchers were limited.

Reporting for specific materials, systems and methods

We require information from authors about some types of materials, experimental systems and methods used in many studies. Here, indicate whether each material, system or method listed is relevant to your study. If you are not sure if a list item applies to your research, read the appropriate section before selecting a response.

Materials & experimental systems

n/a	Involved in the study	
☐	☒	Antibodies
☐	☒	Eukaryotic cell lines
☒	☐	Palaeontology and archaeology
☐	☒	Animals and other organisms
☒	☐	Human research participants
☒	☐	Clinical data
☒	☐	Dual use research of concern

Methods

n/a	Involved in the study	
☒	☐	ChIP-seq
☒	☐	Flow cytometry
☒	☐	MRI-based neuroimaging

Antibodies

Antibodies used	The following antibodies were used in Western blot analysis and co-immunoprecipitation assay: antibodies specific either for EGFR (#4267, 1:1000), p-EGFR (#3777, 1:1000), ERK (#9102, 1:1000), phospho-ERK (#9101, 1:1000), AKT(#9272, 1:1000), p-AKT(#4058,

	1:1000), Grp94 (#20292, 1:1000), Na/K ATPase (#3010, 1:1000), LC3B (#2775, 1:1000), HA (#3724, 1:1000) or p21 (#2947, 1:1000) were purchased from Cell Signaling Technology (MA, USA). Antibodies specific for Grp78 antibody (ab-21685, 1:1000), FBXL2 (ab-153842, 1:1000) were purchased from Abcam (Cambridge, UK). Antibody specific for c-Myc (sc-40, 1:100), Flag (F1804, 1:1000), or TRAF3 (D160776, 1:1000) was purchased from Santa Cruz Biotechnology (CA, USA), Sigma-Aldrich (St. Louis, USA), or Sangon Biotech (Shanghai, China), respectively. Goat anti-mouse IgG-HRP (sc-2005, 1:3000) or goat anti-rabbit IgG-HRP (sc-2004, 1:3000) antibodies was purchased from Santa Cruz Biotechnology (CA, USA). The following antibodies were used in immunofluorescence staining: antibodies specific for EGFR (Cell Signaling Technology, CST-4267, 1:100), Flag (Sigma-Aldrich, F1804, 1:50), or Grp78 (Abcam, ab-21685, 1:200). Rhodamine (TRITC)–conjugated AffiniPure Donkey Anti-mouse IgG (#715-025-150, 1:160) or anti-Rabbit IgG (#711-025-152, 1:160) and Fluorescein (FITC)-conjugated AffiniPure Donkey Anti-mouse IgG (#715-095-150, 1:160) or Anti-Rabbit IgG (#711-095-152, 1:160) were purchased from Jackson Immuno Research (PA, USA). In addition, APC anti-human EGFR antibody (352905, 5μ/Test) and APC Mouse IgG1, κ Isotype Ctrl (FC) antibody (400121, 5μ/Test) were purchased from Biolegend (CA, USA). The following antibodies were used in IHC analyses: antibodies were purchased either from Cell Signaling Technology, Abcam or HUABIO (Hangzhou, China), and specific dilutions were indicated: EGFR (CST-4267, 1:100), p-EGFR (CST-3777, 1:200), FBXL2 (ab-153842, 1:100), HA (CST-3724, 1:1000), Ki67 (Ab-15580, 1:200), cleaved caspase-3 (CST-9661, 1:100) and Cyclin D3 (HuaBio, ET1612-4, 1:50).
Validation	Anti-EGFR (#4267), human and Mouse, WB, IHC and IF, https://www.cellsignal.com/products/primary-antibodies/egf-receptor-d38b1-xp-rabbit-mab/4267; Anti-p-EGFR (#3777), human and Mouse, WB and IHC , https://www.cellsignal.com/products/primary-antibodies/phospho-egf-receptor-tyr1068-d7a5-xp-rabbit-mab/3777; Anti-ERK (#9102), human, WB, https://www.cellsignal.com/products/primary-antibodies/p44-42-mapk-erk1-2-antibody/9102; Anti-phospho-ERK (#9101), human, WB, https://www.cellsignal.com/products/primary-antibodies/phospho-p44-42-mapk-erk1-2-thr202-tyr204-antibody/9101; Anti-AKT(#9272), human, WB, https://www.cellsignal.com/products/primary-antibodies/akt-antibody/9272; Anti-p-AKT(#4058), human, WB, https://www.cellsignal.com/products/primary-antibodies/phospho-akt-ser473-193h12-rabbit-mab/4058; Anti-Grp94 (#20292), human, WB, https://www.cellsignal.com/products/primary-antibodies/grp94-d6x2q-xp-rabbit-mab/20292; Anti-Na/K ATPase (#3010), human, WB, https://www.cellsignal.com/products/primary-antibodies/na-k-atpase-antibody/3010; Anti-LC3B (#2775), human, WB, https://www.cellsignal.com/products/primary-antibodies/lc3b-antibody/2775; Anti-HA (#3724), human and mouse, WB, IP and IHC, https://www.cellsignal.com/products/primary-antibodies/ha-tag-c29f4-rabbit-mab/3724; Anti-p21 (#2947), human, WB, https://www.cellsignal.com/products/primary-antibodies/p21-waf1-cip1-12d1-rabbit-mab/2947; Anti-Grp78 antibody (ab-21685), human, WB and IF, https://www.abcam.com/GRP78-BiP-antibody-ab21685.html; Anti-FBXL2 (ab-153842), huma, WB and IHC, https://www.abcam.com/fbxl2-antibody-n-terminal-ab153842.html; Anti-c-Myc (sc-40), huma, WB, https://www.scbt.com/p/c-myc-antibody-9e10; Anti-Flag (F1804), human, WB, IF and IP, https://www.sigmaaldrich.cn/CN/zh/product/sigma/f1804?context=product; Anti-TRAF3 (D160776), human, WB, https://www.sangon.com/productDetail?productInfo.code=D160776; Anti-Ki67 (Ab-15580), Human and Mouse, IHC, https://www.abcam.com/Ki67-antibody-ab15580.html; Anti-cleaved caspase-3 (CST-9661), Human and Mouse, IHC, https://www.cellsignal.com/products/primary-antibodies/cleaved-caspase-3-asp175-antibody/9661; Anti-Cyclin D3 (HuaBio, ET1612-4), Mouse, IHC, http://www.huabio.cn/product/Cyclin-D3-antibody-ET1612-4; Anti-Goat anti-mouse IgG-HRP antibody (sc-2005), Human, WB, https://www.scbt.com/p/goat-anti-mouse-igg-hrp; Anti-goat anti-rabbit IgG-HRP antibody (sc-2004): Human, WB, https://www.scbt.com/p/goat-anti-rabbit-igg-hrp/; Rhodamine (TRITC)–conjugated AffiniPure Donkey Anti-mouse IgG (#715-025-150), Human, IF, https://www.jacksonimmuno.com/catalog/products/715-025-150; Rhodamine (TRITC)–conjugated AffiniPure Donkey anti-Rabbit IgG (#711-025-152), Human, IF, https://www.jacksonimmuno.com/catalog/products/711-025-152; Fluorescein (FITC)-conjugated AffiniPure Donkey Anti-mouse IgG (#715-095-150) Human, IF, https://www.jacksonimmuno.com/catalog/products/715-095-150; Fluorescein (FITC)-conjugated AffiniPure Donkey Anti-Rabbit IgG (#711-095-152) https://www.jacksonimmuno.com/catalog/products/711-095-152/Donkey-Rabbit-IgG-HL-Fluorescein-FITC; APC anti-human EGFR antibody (352905): Human, FACS, https://www.biolegend.com/en-us/products/apc-anti-human-egfr-antibody-7714?GroupID=BLG9533; APC Mouse IgG1, κ Isotype Ctrl (FC) antibody (400121): Human, FACS,https://www.biolegend.com/en-us/products/apc-mouse-igg1-kappa-isotype-ctrl-fc-3034.

Eukaryotic cell lines

Policy information about cell lines

Cell line source(s)	HEK293T, H1299 and H292 cells were obtained from ATCC. PC-9 cells were obtained from Rio de Janeiro Cell Bank (BCRJ). H1975 cells were provided by Dr. Shengyong Yang (Sichuan University, Chengdu, China) which was originally from ATCC.
Authentication	All cell lines were obtained from a trusted source and were kept at low passages in order to maintain their identity. We have not authenticated these cell lines by ourselves.
Mycoplasma contamination	Cell lines used in this study were routinely tested to be negative for mycoplasma contamination.
Commonly misidentified lines (See ICLAC register)	No commonly misidentified cell lines were used.

Animals and other organisms

Policy information about studies involving animals; ARRIVE guidelines recommended for reporting animal research

Laboratory animals	The Rosa26-Loxp-Stop-Loxp (LSL)-EGFR-L858R/T790M autonomous lung tumor mice were C57BL/6N background and were generated by BEIJING BIOCYTOGEN CO., LTD. Six-week-old male/female Rosa26-LSL-EGFR-L858R/T790M mice were used to treatment with adenoviruses encoding Cre, lentivirus expressing HA-FBXL2 or nebivolol. 6-week-old female BALB/C nude mice were used to treatment with erlotinib, osimertinib, ganetespib, Grp94 inhibitor-1 or nebivolol alone or in combination. Mice were housed in groups of 4–6 mice in an individually ventilated cage (IVC) in a 12:12 light-dark cycle (08:30–20:30 light; 20:30–8:30 dark). The ambient temperature was 22 ± 2°C with 50–60% relative humidity.
Wild animals	No wild animals were employed in the study

Field-collected samples	No field-collected samples were used in this study.
Ethics oversight	All animal care and animal experiments in this study were performed in accordance with the institutional ethical guidelines and were approved by the institutional review board of Sichuan University.

Note that full information on the approval of the study protocol must also be provided in the manuscript.
